# Visual analysis of global research output of lymphedema based on bibliometrics

**DOI:** 10.3389/fonc.2022.926237

**Published:** 2022-08-05

**Authors:** Yun-dong Zhang, Xue Zhang, Xin-yu Wang, Dong-mei Han, Jian-shi Du

**Affiliations:** ^1^ Department of the Lymphatic and Vascular Surgery, China-Japan Union Hospital of Jilin University, Changchun, China; ^2^ Key Laboratory of Lymphatic Surgery Jilin Province, Engineering Laboratory of Lymphatic Surgery, Changchun, China; ^3^ Chengdu Library and Information Center, Chinese Academy of Sciences, Chengdu, China; ^4^ Department of Library, Information and Archives Management, School of Economics and Management, University of Chinese Academy of Sciences, Beijing, China

**Keywords:** lymphedema, cancer-related lymphedema, breast cancer, bibliometrics, data visualization

## Abstract

**Background:**

Globally, several generations of doctors in the field of lymphedema have created numerous publications. To date, no bibliometric analysis has been performed specifically on these publications. For the further promotion of research on lymphedema and to align with the international research frontiers, it is essential to understand the current state of Lymphedema research output.

**Objective:**

This study aims to statistically and visually analyze the characteristics of publications output, distribution of contributions and development process of lymphedema, enriching the knowledge base of Lymphedema, and then seek potential research topics and collaborators.

**Methods:**

Based on the Web of Science core collection database, we firstly analyzed the quantity and quality of publications in the field of lymphedema, secondly profiled the publishing groups in terms of country, institution, author’s publication and cooperation network, and finally sorted out and summarized the hot topics of research.

**Results:**

A total of 8569 papers were retrieved from 1900-2021. The top4 journals with the most publications were LYMPHOLOGY, LYMPHATIC RESEARCH AND BIOLOGY, PLASTIC AND RECONSTRUCTIVE SURGERY and ANNALS OF SURGICAL ONCOLOGY. The top 4 countries with the most publications were USA, Japan, UK, and China. The United States dominates the total number of publications and the international cooperation network. The most productive research institution is Harvard University, and the research institution with the most collaborating institutions is Memorial Sloan Kettering Cancer Center. Mortimer, Peter S contributes the most research in this field. The research achievements of Japanese scholars in this field are of great significance. The top 5 ranked keywords are “Breast Cancer”, “Health-Related Quality Of Life”, “Lymphscintigraphy”, “Lymphovenous Anastomosis”, and “Lymphangiogenesis”.

**Conclusion:**

More and more scholars are devoted to the research of cancer-related Lymphedema. It is foreseeable that breast cancer-related lymphedema and lymphangiogenesis will remain a focus of future research. Advances in Lymphatic vessel imaging and the development of lymphatic microsurgery will further play a role in the clinical workup of lymphedema. Meanwhile, This study can help researchers identify potential collaborators and partner institutions and contribute to further research.

## 1 Introduction

Lymphedema is an acute, transient or chronic progressive disorder of the lymphatic system with insufficiency and disruption of lymphatic transport caused by extrinsic (and/or intrinsic) factors (1). It is characterized by retention of lymphatic fluid, resulting in tissue swelling, which in turn causes proliferative lesions such as tissue fibrosis, fatty deposits and inflammation (2). In general, lymphedema is classified as primary or secondary. Primary lymphedema is caused by congenital lymphatic vessel anomalies. Secondary lymphedema arises as a result of the lymphatic system obstruction or destruction by filariasis, tumors, surgery, trauma and radiation therapy. Treatment for Lymphedema patients includes non-operative treatment based on physiotherapy and surgical treatment based on volume reduction and reconstruction. Frustratingly, these treatments can only alleviate symptoms, delay progress, improve quality of life as much as possible, and cannot achieve the goal of radical cure (3, 4). Lymphedema, an incurable disease that has plagued mankind for hundreds of years, urgently requires researchers to solve this challenge.

Cancer-related lymphedema, which often occurs as a result of lymph node dissection operations secondary to the following cancers: melanoma, head and neck cancer, breast cancer, gynecologic cancer, and urologic cancer, is most common in clinical work of Lymphedema (5, 6). Among them, breast cancer-related lymphedema (BCRL) has been reported most frequently, with the incidence of BCRL being the highest (13% to 65%) and the incidence of cancer-related lymphedema beyond breast cancer being reported in relatively few studies (3, 7–9). The GLOBOCAN 2020 global cancer statistics report showed that there were 19,292,789 new cancer cases worldwide (10). Breast cancer in women became the most common cancer, accounting for 11.7% of the total cases, with approximately 2.3 million new cases worldwide in 2020. The prevalence of BCRL will continue to rise as the number of breast cancer survivors increases due to advancements in detection and treatment technologies. Therefore, it is very important to clarify the pathogenesis, diagnosis and treatment of lymphedema in-depth. A review of lymphedema-related studies shows that few studies try to summarize the development track and trend of Lymphedema, or visualize the current research hotspots in this field. However, these studies provide an important forward-looking reference value for timely grasping the whole picture of the development of Lymphedema and distinguishing the defects and deficiencies of the current research.

Bibliometrics takes documents, books and other communication media with different data sources as the research object, and uses deterministic metrological methods such as mathematics and statistics to analyze the metrological characteristics of the research object, revealing and studying the quantitative relationship, distribution structure, changing rules and research hotspots of article and information, and then describe, evaluate and predict the current situation and development trend of science and technology (11, 12). Social network is a collection of social actors and their relationships, and social network analysis method is a set of norms and methods to analyze the structure of relationships and their properties of social networks by using mathematics, statistics, graph theory and other integrated quantitative analysis tools.

Based on this, the study took the articles related to lymphedema as the research object and used bibliometric analysis method and social network analysis method to analyze the characteristics of publication output and the evolution trend of cooperation network. Our primary objectives were as follows: (i) we aimed to clarify the publication output and influence of lymphedema research; (ii) from the macro, meso, and micro levels, we aimed to analyze the publication situation, evolution trend, and cooperation network of countries, institutions, and authors in this field, respectively; and (iii) we aimed to summarize the research topics/hotspots in this field. With the help of the above analysis, we hope that this study will provide a panorama of knowledge on the historical development of Lymphedema, so that it provide data to support the search for potential research gaps between countries and institutions; provide a knowledge base of relevant article for different research topics and lay out research priorities in advance; and then provide directions for selecting research topics, identifying potential collaborators and obtaining research funding support.

## 2 Data sources and study methods

### 2.1 Data sources

The Web of Science database of Clarivate Analytics is the most authoritative indexing tool for scientific and technological article in the world. It is an integrated platform of multiple databases, providing the most important research results. Besides, the index and archives can be traced back to 1900. The Web of Science Core Collection Database is an important sub-database of the Web of Science and an important database for obtaining global academic information. It contains more than 20000 authoritative and influential academic journals around the world, covering natural science, engineering technology, biological science and other fields. At the same time, the Web of Science core collection includes references cited in papers, and a unique citation index is compiled according to the cited author, source and publication year. Its comprehensiveness, authority and universality have been widely recognized by researchers^^1^^

**Figure 1:**
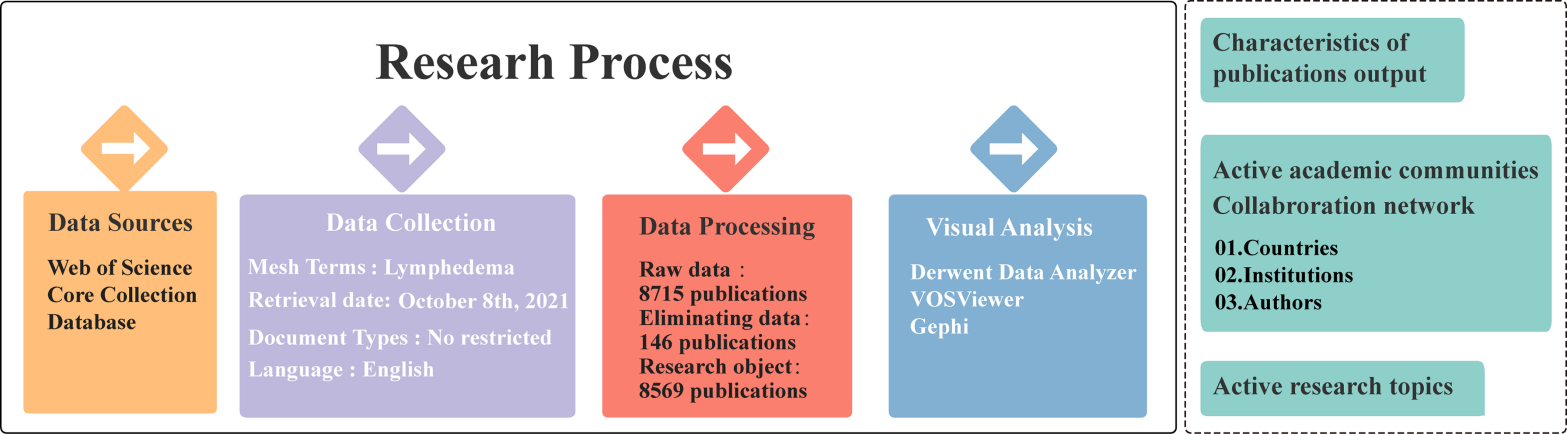
The detailed integrated workflow.

Through in-depth and comprehensive retrieval of the database resources, we can effectively trace the historical context of a certain subject field since the first publication was issued, to find a highly influential article collection and locate the current research focus. Based on the above analysis, this study takes the Web of Science core collection as the data source. The specific retrieval strategy is shown in **Figure 1**: Mesh term of lymphedema is used as topic words; The time span is no restricted, the retrieval time is October 8, 2021; and a total of 8,715 journal articles have been retrieved. Through manual discrimination, the above-mentioned articles were screened and deduplicated, and a total of 146 articles that were irrelevant to this research field or missing key fields were eliminated, and finally 8569 articles to be analyzed were obtained. The following will take 8569 articles as research samples. Besides, bibliometrics and other research methods are used for analysis to gain insight into the development trends and research hotspots in the field of lymphedema. The integrated workflow is detailed in **Figure 1**.

### 2.2 Analysis strategy

Firstly, this study analyzes the general overview and its citation influence of scientific research output in the field of lymphedema, laying a foundation for researchers to gain insight into the overall picture of this field. Secondly, from the macro, meso and micro levels, this paper analyzes the publication, evolution trend and cooperation network of countries, institutions and authors in this field. Furthermore, it also explores the core countries, institutions, authors and groups with more frequent cooperation relations in the cooperation network. Then researchers can grasp the core countries, institutions, authors and their research focuses in this field from multiple levels and dimensions, and provide references for searching and reading high-quality articles on different topics. Finally, this study summarizes the research topics in this field, and provides support for researchers to grasp the current research hotspots, predict the future evolution trend of the topic, and then lay out the research directions with forward-looking value in advance.

### 2.3 Research method

Based on the above analysis process, the main methods used in this study are as follows:① Bibliometric analysis method, the main tool used is Derwent Data Analyzer (DDA, Clarivate, USA). DDA is a powerful text analysis tool developed by Clarivate Analytics, which can carry out multi angle data mining and visual panoramic analysis on text data, especially carrying out data cleaning and analysis on the source data of the Web of Science platform. In this study, DDA is first used for data preprocessing. The journals, countries, institutions, authors and keywords of 8569 documents are standardized (for example, the same institution has different writing forms in different documents, but the keywords in different writing forms have the same meaning, etc.). Secondly, word frequency statistical analysis is carried out to generate a co-occurrence matrix of high-frequency countries, institutions, authors and keywords, which lays a data foundation for the following social network analysis and visualization. ② Social network analysis method, the main tool used are VOSviewer (VOSviewer,Leiden University, Netherlands) and (Gephi, WebAtlas, France). VOSviewer is a free visualization tool for social network analysis developed by Leiden University in the Netherlands. It can visually analyze document citations, keywords, cooperation, etc., and can interpret the strength and interaction of different groups through the color, size, clustering results and other information of nodes. Compared with other visualization software, the greatest advantage lies in its powerful graphic display ability, and its map effect is accurate and exquisite. However, the nodes cannot be dragged at will, and if there are many nodes, it is easy to block them, so the readability is reduced. Correspondingly, another visualization tool Gephi is an open source and free cross platform complex network analysis and data visualization tool based on JVM. It is embedded with a variety of layout, statistics, filtering and other algorithms, which can convert network data into visual image data. Based on the results of DDA data preprocessing, VOSviewer and Gephi were used to draw the visualization maps of high-frequency countries, institutions, authors and keywords respectively. Then, the readability and interpretability of the two maps were comprehensively compared, and the maps which can better reflect the actual situation were selected.

## 3 Results

### 3.1 Characteristics of lymphedema publication output

#### 3.1.1 The document types included in the study

**Figure 2:**
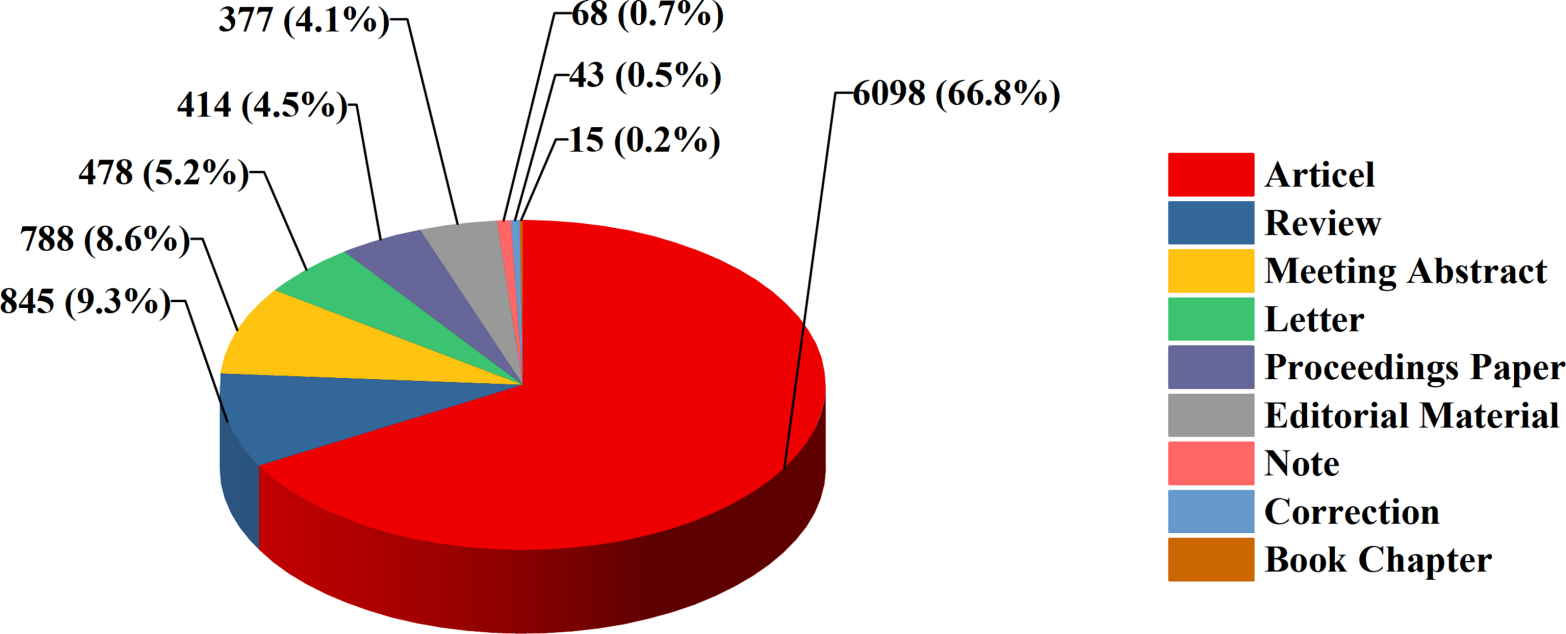
Lymphedema research papers by document type percentage.

We took the 8569 publications related to lymphedema in the Web of Science Core Collection from 1900 to 2021 as the research object. These studies could be classified into 9 study types. Original articles (66.8%) were the most dominant type of publications throughout the whole period, and reviews accounted for 9.3% of articles (**Figure 2**).

#### 3.1.2 The trends of annual publication volume and cited frequency

**Figure 3:**
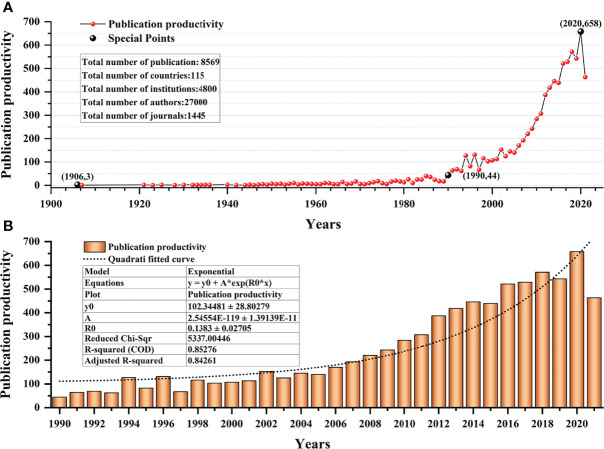
The publication volume and cited frequency on the topic of lymphedema. **(A)** The publication volume of lymphedema from 1900 to 2021. **(B)** The publication volume of lymphedema from 1990 to 2021.

By analyzing the publication year of 8569 publications, the publication output trends of lymphedema were clarified. From 1900 to 8 October 2021, the volume and cited frequency of publications showed a continuously increasing trend. In terms of the volume of publications, the number of publications on lymphedema research increased steadily from 1900 to 1990 and increased rapidly from 1990 to 2021; 44 studies were published in 1992, and 658 were published in 2020; the volume of publications in 2020 was nearly 15 times that in 1992. It shows that the growth trends of the volume of publications have increased exponentially over the past 30 years (**Figure 3**). According to the statistics of 8569 cited articles, up to October 8, 2021, there were 69120 cited articles, and the cited frequency was 188072 times. Additional details of the citation analysis are described in **Table 1**.

**Table 1:** The results of citation analysis of 8569 articles.

Citing articles	Without self-citations	Sum of times cited	Without self-citations	Average citations per item	h-index
69120	62203	188072	113117	21.56	156

#### 3.1.3 Distribution of publications by journal

A total of 8569 publications were published in 1445 different journals. Four journals (LYMPHOLOGY, LYMPHATIC RESEARCH AND BIOLOGY, PLASTIC AND RECONSTRUCTIVE SURGERY, and ANNALS OF SURGICAL ONCOLOGY) had the highest number of published papers, each accounting for more than 2% of the total publications; altogether, the publications published in these four journals accounted for 15% of the total publications. **Supplementary Figure S1** shows a total of 23 unduplicated journals, which are clearly of great value for research on the subject of lymphedema. Therefore, highlighting articles from these major journals helps us to keep up with the latest trends.

#### 3.1.4 Distribution of publications by discipline categories

At present, the Web of Science database includes 256 different disciplinary categories, and an analysis of the discipline categories of 8569 publications on lymphedema indicated that scholars in many disciplines are focusing on this topic; And the publications were distributed in 126 discipline categories. **Table 2** shows that the discipline category with the most publication output was oncology, followed by the remaining top 5 discipline categories in the following order: surgery, physiology, immunology, dermatology, medicine, research & experimental. In addition, all the discipline categories of the above-mentioned 23 journals were included in these 15 discipline categories without exception.

**Table 2:** The discipline category with the most publication output.

Rank	Discipline categories of WoS	Frequency (N)	Percentage (%)
1	Oncology	2032	15.18%
2	Surgery	1975	14.75%
3	Physiology	1036	7.74%
4	Immunology	672	5.02%
5	Dermatology	612	4.57%
6	Medicine, Research & Experimental	606	4.53%
7	Medicine, General & Internal	490	3.66%
8	Radiology, Nuclear Medicine & Medical Imaging	489	3.65%
9	Obstetrics & Gynecology	462	3.45%
10	Peripheral Vascular Disease	449	3.35%
11	Rehabilitation	364	2.72%
12	Genetics & Heredity	298	2.23%
13	Health Care Sciences & Services	242	1.81%
14	Nursing	212	1.58%
15	Cell Biology	193	1.44%

#### 3.1.5 Distribution of the top 100 highly cited publications

**Figure 4:**
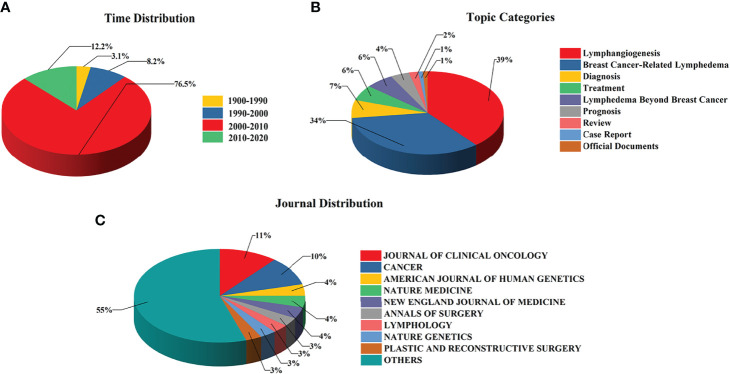
The research output characteristics of the top 100 highly cited publications. **(A)** The time distribution of top 100 highly cited publications. **(B)** The topic categories of top 100 highly cited publications. **(C)** The journal distribution of top 100 highly cited publications.

We retrieved 6,649 publications out of 8,569 publications that were cited more than once, accounting for 77.6% of all publications. We examined the top 100 cited publications (cited 198 times and above) in terms of time distribution, research topics, and journal sources (**Supplementary Table S2**). The details are shown in **Figure 4**. Among them, the largest number of publications (76.5%) was obtained from 2000 to 2010, and the topics of the top 100 cited publications were mainly Breast Cancer-related Lymphedema and Lymphangiogenesis. Among these publications, the most frequently cited articles are published in nine different journals; JOURNAL OF CLINICAL ONCOLOGY had the highest number of articles (11%) followed by CANCER (10%) and NATURE MEDICINE (5%).

### 3.2 Major academic communities of lymphedema publications

We collected information on 8569 publications discussing lymphedema and extracted the country, institution, and author entries contained therein to present the main academic groups on lymphedema at the macro, meso, and micro levels, respectively.

#### 3.2.1 Distribution of publication output by countries

**Figure 5:**
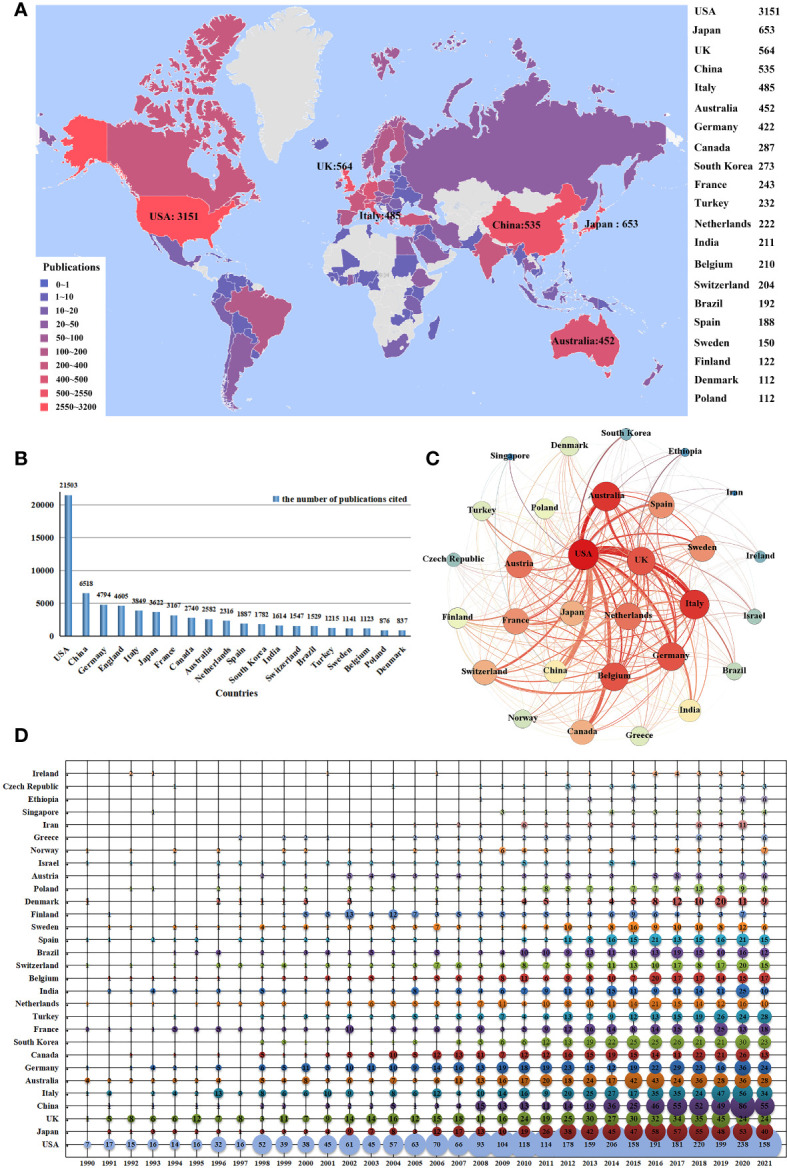
The distribution of publication output by countries. **(A)** The spatial distribution of top 20 most productive countries producing lymphedema publications. **(B)** The top 20 citing countries on lymphedema. **(C)** The cooperation network of co-publication of lymphedema publications among selected countries. **(D)** Temporal distribution of top 30 most productive countries of lymphedema publication.

The total number of publications in the field of lymphedema covers 114 different countries. **Figure 5A** shows the distribution of countries with more than or equal to 112 publications using the software FineBI. The USA contributed the highest number of publications (n=3151, 31.56%; n indicates the number of publications published; the percentage indicates the percentage of the number of publications published in that country to the total number of publications published thereafter) followed by Japan (n=653, 6.54%), the United Kingdom (n=564, 5.65%), and China (n=535, 5.36%); these four countries had a total share of 49.11%. **Figure 5B** shows that USA scholars cited the highest number of article in the field of lymphedema (n=21503, n indicates the number of articles cited).

Further analysis of the temporal distribution of the top 20 most productive countries in terms of lymphedema papers showed that USA, Japan, UK, Italy, Australia, and Germany are the leaders in this research area. Notably, the number of publications from China, Korea, and India has grown rapidly since 2010 (**Figure 5D**).

We selected 30 countries with more than 10 publications to build a national cooperation network map (**Figure 5C**). USA academic institutions and organizations (as the main co-author partners of international co-authorship) formed a cooperation network with the USA at the core. USA had particularly close collaborative relationships with institutions/organizations in the field of lymphedema, including those in the UK (n=112; n indicates the number of co-authors), China (n=99), Australia (n=75), Italy (n=69), Canada (n=69), Germany (n=59), and Japan (n=54). Strong and stable collaborative relationships were also formed among the UK, Italy, Australia, and Germany.

#### 3.2.2 Distribution of publication output by institutions

**Figure 6:**
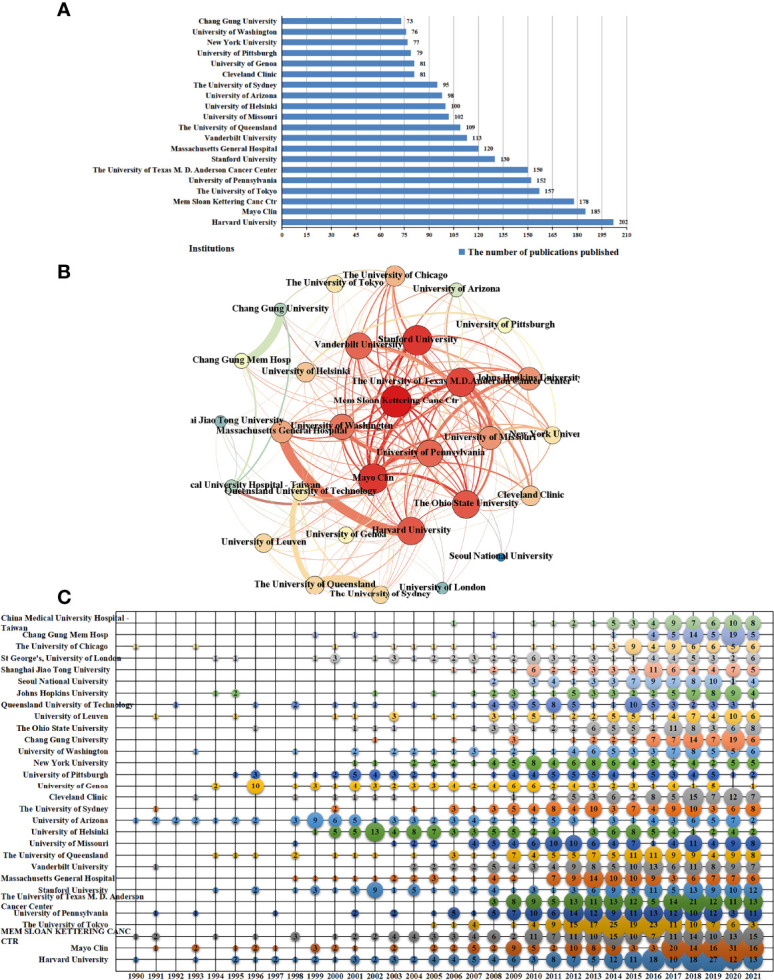
The distribution of publication output by institutions. **(A)** The top 20 most-productive institutions of lymphedema publications. **(B)** The co-authorship network of lymphedema publications among principal research institutions. **(C)** The temporal distribution of top 30 most-productive institutions of lymphedema publications.

A count of the institutions publishing 8569 publications showed that more than 4800 institutions/organizations were involved in this area of research. We analyzed the number of papers published by the top five institutions shown in **Figure 6A** and found that these institutions/organizations accounted for 10.2% of the total papers. **Figure 6C** shows the top institutions that have been publishing since 1990, as follows: Harvard University, Mayo Clin, Mem Sloan Kettering Canc Ctr, and University of Arizona. The top three institutions have maintained high publication volumes in recent years. The University of Tokyo and The University of Texas M.D. Anderson Cancer Center showed a rapid increase in the number of publications in recent years and maintained high publication levels.

Collaboration among organizations/institutions was a relatively common phenomenon, as shown in **Figure 6B**. Chang Gung University and Chang Gung Memorial Hospital, Harvard University and Massachusetts General Hospital, and The University of Queensland and The University of Sydney had the highest number of collaborative publications. Moreover, a collaborative network relationship was formed with Memorial Sloan Kettering Cancer Center at the core.

#### 3.2.3 Distribution of publication output by authors

Statistically, this study involved more than 27,000 different authors, of which the number of authors who published only one paper was approximately 21,000, thereby accounting for approximately 77% of the total number of authors. **Table 3** lists the top 20 authors in terms of the number of publications; the number of publications of the top 10 authors accounted for 10% of the total number of publications.

**Table 3:** The top 20 authors in terms of the number of publications.

Rank	Authors	Frequency	Affiliations	Percentage
1	Mortimer, Peter S	128	St Georges University London	1.49%
2	Koshima, Isao	106	University of Tokyo	1.24%
3	Yamamoto, Takumi	103	University of Tokyo	1.20%
4	Campisi, Corrado	95	University of Genoa	1.11%
5	Boccardo, Francesco	94	University of Genoa	1.10%
6	Rockson, Stanley G	94	Stanford University	1.10%
7	Armer, Jane M	82	University of Missouri System	0.96%
8	Ridner, Sheila Hedden	81	Vanderbilt University	0.95%
9	Mehrara, Babak J	78	Memorial Sloan Kettering Cancer Center	0.91%
10	Witte, Marlys H	75	University of Arizona	0.88%
11	Taghian, Alphonse G	72	Harvard University	0.84%
12	Mihara, Makoto	65	University of Tokyo	0.76%
13	Olszewski, Waldemar Lech	63	Polish Academy of Sciences	0.74%
14	Narushima, Mitsunaga	61	University of Missouri System	0.71%
15	Hara, Hisako	57	University of Tokyo	0.67%
16	Kilbreath, Sharon Lynn	57	University of Sydney	0.67%
17	Chang, David W	56	University of Chicago	0.65%
18	Cheng, Ming-Huei	54	Chang Gung Memorial Hospital	0.63%
19	Schmitz, Kathryn H	54	University of Pennsylvania	0.63%
20	Ward, Leigh C	52	University of Queensland	0.61%

**Figure 7:**
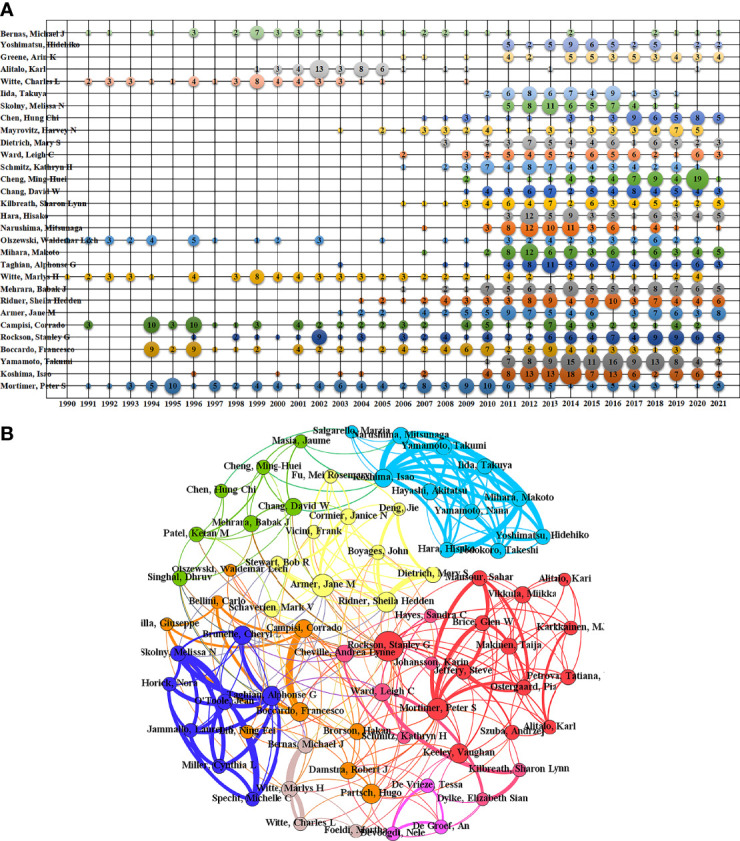
The distribution of publication output by authors. **(A)** The temporal distribution of top 30 most-productive authors of lymphedema publication. **(B)** The co-author networks of lymphedema publications.

**Figure 7A** clearly shows the trend in the number of publications of the top 30 authors most productive over time since 1990. “Mortimer, Peter S”, “Boccardo, Francesco”, “Rockson, Stanley G”, “Campisi, Corrado”, “Witte, Marlys H”, “Olszewski, Waldemar Lech” continued to show a high output in the field of lymphedema since 1990. Many rising stars have emerged in the field since 2000, such as “Armer, Jane M”, “Ridner, Sheila Hedden”, “Mehrara, Babak J”, and others. In the past 10 years, Koshima, Isao, Yamamoto, Takumi, and others have produced more publications, and “Witte, Charles L”, “Alitalo, Karl”, “Bernas, Michael J” and others used to publish more publications. However, there was almost no output in this field in recent years.

The 104 authors with more than 20 publications were selected to construct the author collaboration network, and five authors were screened out because they did not produce collaboration with other authors (**Figure 7B**). Scholars, such as “Koshima, Isao”, “Yamamoto, Takumi”, “Campisi, Corrado” and “Boccardo, Francesco”, collaborated more frequently than the others. Further analysis was performed by using a modularity algorithm to divide the authors into different collaborative subgroups to visualize the small groups with frequent interactions. The entire collaborative network is divided into nine more distinct subgroups.

### 3.3 Keywords and hotspots of lymphedema publication output

#### 3.3.1 The frequency network of keywords about lymphedema publications

**Figure 8:**
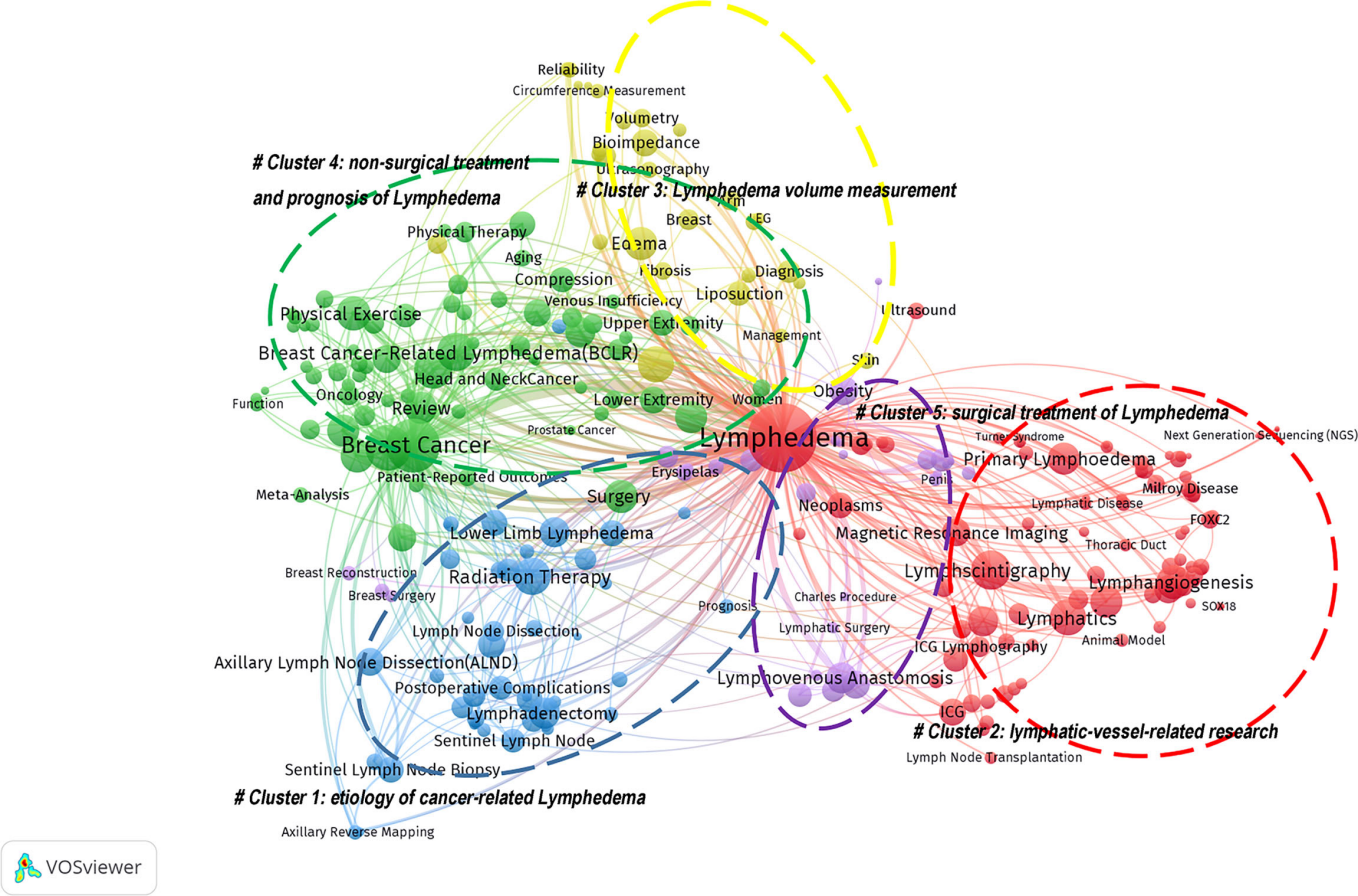
The cluster network based on Lymphedema high-frequency keywords (≥10 times). Cluster1 (Blue circle): research on the etiology of cancer-related Lymphedema; Cluster 2 (Red circle): lymphatic-vessel-related research, including the main terms of lymphangiogenesis and lymphatic system imaging; Cluster 3 (Yellow circle): Lymphedema volume measurement; Clusters 4 (Green circle): research on non-surgical treatment and prognosis of Lymphedema; and Clusters 5 (Purple circle): studies on the surgical treatment of Lymphedema.

A total of 7487 different keywords were involved in this study, and 6908 keywords remained after cleaning and merging through the following: deactivating words; combining cases, singular, and plural; lexical reduction; and finding stems. We counted 261 keywords that appeared at least 10 times in publications on the topic of lymphedema (**Supplemental Table S3**). The top five keywords in order are “Breast Cancer”, “Health-Related Quality of Life”, “Lymphscintigraphy”, “Lymphovenous Anastomosis” and “Lymphangiogenesis”. A total of 261 keywords were clustered and analyzed by VosViewer through the steps of data format conversion and keyword co-citation matrix construction. **Figure 8** shows that research in the field of lymphedema mainly focused on five areas.

#### 3.3.2 The analysis of representative keywords

**Figure 9:**
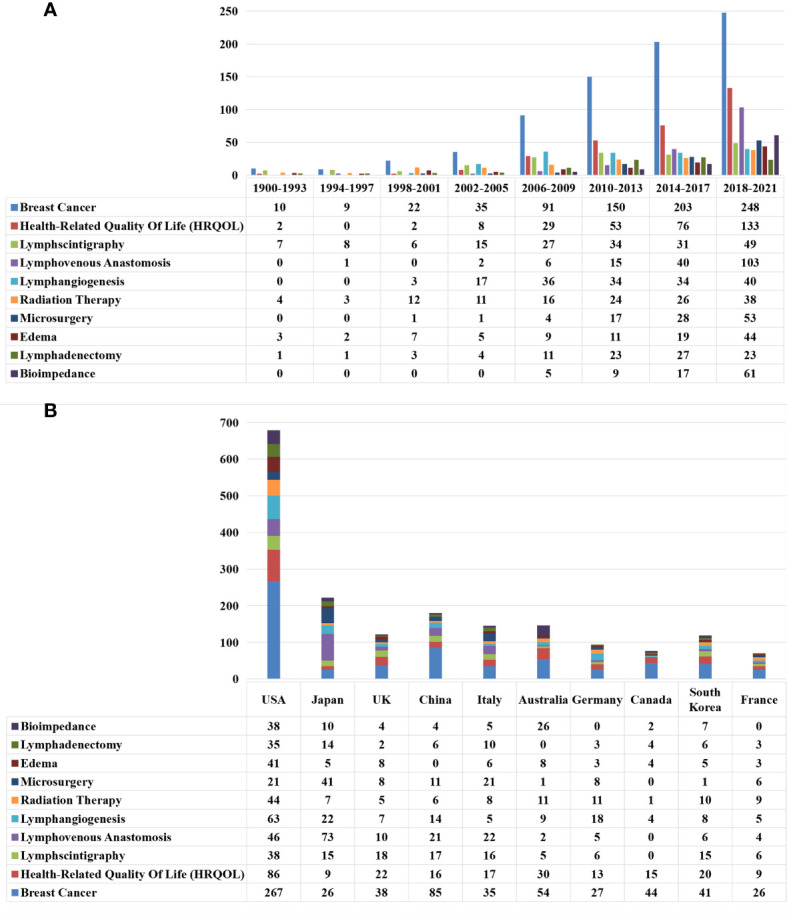
The Characteristics of the top-ranked representative keywords in the 5 clustered topics. **(A)** The annual number of new publications of the top-ranked representative keywords in the 5 clustered topics. between 1900 and 2021. **(B)** The distribution of the number of top-ranked representative keywords in the 5 clustered topics among the top 10 countries producing lymphedema publications.

We selected the top-ranked representative keywords in the five clustered topics, and the number of publications for these keywords per year since 1990 is shown in **Figure 9A**. The number of studies on lymphedema with the keywords “Breast Cancer”, “Health-Related Quality of Life “, and “Lymphovenous Anastomosis” was high, showing a continuous and steady level of growth. The number of studies with the keywords “Lymphovenous Anastomosis” and “Bioimpedance Spectroscopy” increased rapidly after 2014. The distribution of the focus studies in the top 10 countries for these keywords is shown in **Figure 9B**. The number of studies on lymphedema with “Breast Cancer” as a keyword was high in each country, especially in the USA, China, and Australia. Studies on lymphedema with the keyword “Health-Related Quality of Life” were more in the USA, United Kingdom, Australia, and South Korea. Japan had the highest number of studies on lymphoedema with the keywords “Lymphovenous Anastomosis” and “Microsurgery”. USA had the highest number of studies with the other eight keywords.

## 4 Discussion

In this study, we combined bibliometric analysis with network visualization to describe the current state of lymphedema research; analyze the characteristics of journal output and the contributions of countries, institutions, and authors in this field; and introduce the hotspots of lymphedema science research. Through analysis, we visualized the following development trends of lymphedema in terms of the characteristics of publication output, major academic communities, and active research topics.

### 4.1 Research output characteristics of lymphedema publications.

Since 1900, the annual publications output in this field increased steadily. In the past few decades, the related scientific publications output expanded explosively; the number of publications accounted for 58.06% of all published works in the past decade. The growth trend of the citation frequency of articles published in this field was similar to the annual publication output volume. And the research of lymphedema has multidisciplinary and interdisciplinary trends. All information showed that scientific research on lymphedema is widely recognized and continues to be a popular topic in the academic community. Combined with the number of journal publications, citations, and journal subject categories, the development of oncology surgery has an absolute advantage and influence on the research output. On the one hand, this finding is due to the high incidence of cancer-related lymphedema and the increase in researcher investment (3, 8, 10). On the other hand, the development of microsurgery has further promoted the progress of lymphedema research (13–17).

### 4.2 Major academic groups exhibit different characteristics at different levels.

#### 4.2.1 National level:

Original articles are the most valuable publications; these occupy a high position in evidence-based medicine. The number of original papers a country published best reflected its research productivity. The level of scientific research development and the number of publications were proportional to the country’s GDP. The USA, as the world’s largest economy, was the center and leader of the network of co-authored papers about lymphedema. It had the largest number of publications and citations. These results suggested that the USA has a significant impact on the direction of lymphedema research in the most powerful cooperative network around the world. It is worth noting that Japan, which ranked second in the world in lymphedema research output, contributed to a large number of scientific research on lymphedema, but its partnerships were mainly domestic. China ranked fourth in the number of papers published in the world, second in citation papers, and sixth in cooperation with other countries. China’s article output grew exponentially. The output of articles from South Korea and India also grew steadily. This growth over the past two decades may be due to the huge economic development in these Asian countries. The number of articles in European countries has also maintained a steady growth, and co-author analysis shows that the main cooperation networks are still carried out with developed countries at the core.

#### 4.2.2 Institutional level:

In addition to economic conditions, several other factors determined the number and quality of publications, such as the number of inhabitants in each country, the number of lymphedema doctors in different countries (unfortunately, these data are not available to us), and the level of research development in medical schools or institutions. Statistics showed that 70% of the top 20 institutions were located in the USA, indicating that the USA had excellent traditional research institutions. Harvard Medical School, the oldest medical institution in the United States, was the most productive research institution in terms of lymphedema publications. In addition, Memorial Sloan Kettering Cancer Center ranked first in the agency’s co-author analysis, indicating that it worked closely with other agencies, which is in line with the purpose of their organization; collaboration is a hallmark of the research enterprise at The Sloan Kettering Institute. The University of Tokyo in Japan ranked fourth in productivity but not very prominently in the co-authorship analysis, thereby exhibiting unique self-creativity and contribution. The co-authorship network of research institutions showed significant collaboration between the two domestic institutions, except for the co-authorship network of USA institutions.

#### 4.2.3 Author level:

Through the statistics of the author’s lymphedema research paper information, we determined the active researchers and academic leaders and whether they formed an excellent research team to a certain extent. Unlike the co-authorship network structure of countries and institutions, the co-authorship analysis among different authors was divided into multi-center co-authorship network instead of the network structure of developed countries, such as the network with the USA as the main core. The different co-authorship network structures showed a relatively centralized research topic. The nine more notable co-author groups in the current study were as follow: (i) the group with the core of Rockson, Stanley G on the study of genetic mechanisms of lymphatic system diseases and lymphedema (18, 19); (ii)Ward, Leigh C, Cheville, Andrea Lynne on the study of application of bioimpedance analysis in breast cancer-related lymphedema (20–22); (iii) the trio group with Devoogdt, Nele et al. on the study of the application of questionnaires to upper limb lymphedema (23, 24); (iv) the group with the core of Partsch, Hugo on the study of the application of microsurgery to breast cancer-associated lymphedema (25, 26); (v) the group with the core of Witte, MH, whose research topic was FOCX2 in primary lymphedema (27, 28); (vi) the group with the core of Taghian, Alphonse G, whose study topic was risk factors for lymphedema secondary to breast cancer (29–33); (vii) the group with the core of Chang, David W and Cheng, Ming-Huei whose study topic was surgical treatment of vascularized lymph node transfer (34, 35); (viii) the group with the core of Armer, Jane M, whose study topics were head and neck cancer-associated lymphedema, prognosis of cancer-associated lymphedema, physical therapy and care (36–38); (ix) the group with the core of Koshima, Isao, whose research themes were applied research of indocyanine green (ICG) and lymphovascular venous anastomosis (39–43). Among them, the Japanese research scholars, with Koshima Isao at the core, constituted the closest co-authorship network structure, and their research themes are the most explicit. This finding further confirmed that Japan had the strongest creativity in the distribution of lymphovenous anastomosis among different countries. Japanese scholars published articles on the subject of lymphedema, as follows: (i) practical experience of ICG imaging in the field of lymphedema (44); (ii) application of ICG as an intraoperative NIR fluorescent imaging marker (45); (iii) staging of lymphedema based on ICG (46, 47); and (iv) further investigation of lymphovenous anastomosis under the guidance of ICG imaging (48). The great contributions of Japanese scholars to the advancement of microscopic instruments and the development of microsurgery have led to research advances in the field of lymphedema.

Co-authorship represents the most formal form of intellectual cooperation in the field of science. When two or more authors collaborate on research, more scientific output and higher quality studies can be obtained. The co-authorship in the field of lymphedema needs to be strengthened in the future among various countries and institutions. If it was possible, we would be able to choose our partners from the established Lymphedema academic teams.

### 4.3 The research hotspots in the field of lymphedema

Keywords provide important information about research trends and frontiers and reveal the areas of research interest. The analysis of this study identified five main areas of research focus in the field of lymphedema, as follows: research on the etiology of cancer-related Lymphedema (Cluster1); lymphatic-vessel-related research (Cluster 2), including the main terms of lymphangiogenesis and lymphatic system imaging; Lymphedema volume measurement (Cluster 3); research on non-surgical treatment and prognosis of Lymphedema (Clusters 4); and studies on the surgical treatment of Lymphedema (lusters 5).

#### 4.3.1 Cluster1: The etiological study of cancer-related lymphedema

With the increasing incidence of cancer, more and more patients are undergoing multimodal treatment, including surgery, radiotherapy, chemotherapy and hormone therapy. Cancer treatments involving lymph nodes can disrupt lymphatic drainage, which can lead to the accumulation of lymphatic fluid in the interstitial tissues of the involved limbs and body regions and result in secondary lymphedema (3). In terms of BCRL, the incidence of BCRL is about 33% to 47% after axillary lymph node dissection (ALND) and radiation therapy, and about 4% to 17% after sentinel lymph node biopsy and radiation therapy (49). Although the treatment of cancer-related lymphedema can be effective in the short term, the long-term results may be poor or difficult to maintain. Therefore, we should not only strengthen the etiological prevention, but also avoid damaging the lymphatic drainage pathway or reconstructing the physiological lymphatic drainage channel in the first stage (50).

Kimberg introduced the concept of axillary reverse mapping (ARM) in 2007 (51). ARM is a preoperative technique designed to protect the ipsilateral arm-to-axillary lymphatic channel during surgical intervention for breast cancer (52). The most frequently cited publication in this study was “LYMPHATIC MAPPING AND SENTINEL LYMPHADENECTOMY FOR BREAST-CANCER” (GIULIANO, AE; KIRGAN, DM; GUENTHER, JM; MORTON, DL et al., 1994), with a total of 2007 citations and an average annual citation of 71.68. This study accurately identified the anterior lymph nodes in certain patients by intraoperative lymphatic labeling, thereby improving the accuracy of staging while altering the disruption of lymphatic channels by ALND (49). A meta analysis involving 1659 patients recorded the incidence of lymphedema (53). A total of 37 patients suffered arm lymphedema (37/786, 4.71%) in the experimental group (ARM during ALND), compared with 164 arm lymphedemas (164/873, 18.79%) in the control group (ALND alone) (53). In addition, the oncological safety of the ARM method has been questioned; reliable and convincing prospective studies to assess the efficacy and oncological safety of the ARM method are still underway (7).

Prophylactic lymphovenous anastomosis, also known as immediate Lymphatic reconstruction (ILR), is a method that allows the technique of lymphovenous anastomosis to be performed at the time of cancer resection with lymph node dissection to reduce the risk of lymphedema (54, 55). In essence, it is the spatio-temporal transformation of lymphovenous anastomosis from the treatment of Lymphedema to the prevention of Lymphedema (56). In recent years, the possibility of prophylactic lymphovenous reconstruction guided by ICG imaging has become feasible. Several recent systematic evaluations and meta-analyses have shown that IRL can effectively reduce the incidence of lymphedema in patients after lymph node dissection (57, 58). As a result, in order to reduce the incidence of lymphedema, IRL brings greater hope for cancer patients proposed for sugery.

#### 4.3.2 Cluster 2: The lymphatic-vessel-related research

##### 4.3.2.1 Lymphangiogenesis

As shown in our analysis of the top 100 highly cited article, the most represented topic was lymphangiogenesis-related mechanistic studies. In addition, “Lymphangiogenesis” with 164 word frequency. VEGF-C/VEGFR3 signaling plays a key role in the generation, development and maturation of lymphatic vessels. Other more studied genes or molecules found in this analysis include Foxc2, Gata2, Prox1, Flt4, Sox18, LYVE-1, etc (7, 59). Of course, not limited to the molecules shown in our analysis, other important mechanisms and molecules include the matrix protein ccbe1, the metalloprotease Adamts3, neuropilin2, b1-integRin, Celsr1, Vangl2, Pkd1, Pkd2, and Fat4, Rasip1, Orai1 and Piezo1, Wnt/b-catenin Signaling, PDGFB, etc. (59). In a recent study, Abumrad, Nada A et al. found that CD36 deficiency in LECs leads to obesity and leaky intestinal lymphatics; CD36 regulates oxidative metabolism and function of Lymphatic endothelial cells (LECs) *in vitro*. CD36 deficiency in LECs highlights a novel mechanism for the etiology of visceral obesity and insulin resistance. Considering the important function of the lymphatic system in tissue homeostasis, LEC CD36 is also may be associated with lymphedema disease (60). The study of the cellular and molecular mechanisms of lymphangiogenesis is crucial to understand the origin of human lymphatic disorders and will provide new ideas for the development of new therapeutic agents for the treatment of these pathological diseases. Now, at the cellular and molecular levels, Hwang, Ji Hye et al. demonstrated the significant effect of VEGF-C to enhance lymphangiogenesis by human adipose stem cells in combination with VEGF-C hydrogel in a mouse model of lymphedema (61). In clinical trials, the safety of the combination of lymph node metastasis and adenoviral VEGF-C was demonstrated in a small trial of lymphedema associated with breast cancer (62). Danish research groups pioneered clinical trials of adipose-derived regenerative cells for lymphedema, and in 2016, Toyotaserkani et al. reported a pilot study using adipose stem cells adjuvant fat transfer for lymphedema, where 4 weeks after treatment, patients’ daily arm heaviness, tension symptoms were significantly reduced, the number of compression treatments was decreased, the volume of the affected limb was reduced, and no postoperative complications or adverse events occurred; and they were conducting a phase III clinical trial (63, 64). As discussed above, a better understanding of how different genes or molecules affect the normal function of the lymphatic vascular system and the identification of new pro-lymphopoietic factors will help in the diagnosis and eventual treatment of patients with symptomatic or asymptomatic lymphatic disorders and related comorbidities.

##### 4.3.2.2 Lymphatic-vessel imaging

**Figure 10:**
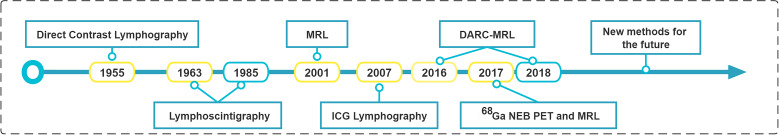
The historical progress of Lymphatic-vessel imaging.

The historical progress of Lymphatic-vessel imaging is shown in **Figure 10**. Direct or indirect imaging of the lymphatic system is still the most valuable reference for the evaluation of lymphedema and the choice of operation. Regarding lymphatic vessel imaging, the top word frequency is “Lymphscintigraphy”, n=179. Since 1963, Lymphscintigraphy has been widely regarded as the gold standard for confirming the diagnosis of lymphedema, showing the functional status of the lymphatic system, and as the primary test to guide clinical management. However, it cannot show tissue edema and fibrosis around lymphedema, and suffers from isotopic contamination. Although Lymphscintigraphy is highly sensitive, it lacks standardization (65, 66). The Lymphscintigraphy we use today is mainly a combination of ^99m^Tc-labeled colloid and gamma scintillation camera imaging modality (67, 68). Because of the low resolution issues, the development of combined multimodal imaging modalities, such as ^68^Ga-labeled Evans Blue (^68^Ga-NEB) PET imaging in combination with magnetic resonance lymphangiography (MRL), has shown significant advantages in assessing the severity of lymphedema, staging, and site of lymphadenopathy for individualized treatment planning (66, 69). In addition, Ming-Huei Cheng, et al.’s Taiwan lymphoscintigraphy staging was validated with cheng’s lymphedema grading and ICG imaging to provide practical guidelines for lymphedema grading and treatment (65, 70, 71). In recent years, MRL is routinely used for preoperative planning of lymphedema surgery because its high-resolution display of fine vascular structures may improve the outcome of lymphedema surgery (72). Recent studies have demonstrated that the absence of venous signals in Dual-Agent Relaxation Contrast MRL has the potential to further improve the efficiency and accuracy of MRL (73, 74). With the development of near-infrared imaging technology and the introduction of ICG, indirect lymphatic system imaging with ICG has been widely used in clinical practice (44, 45, 75). Japanese scholars have made the staging of upper limb, lower limb and genital area of secondary lymphedema based on ICG lymphography (46–48). They divided the ICG lymphography performance into two types: normal line type and abnormal dermal reflux type. In conclusion, MRL and ICG lymphangiography bring more possibilities for lymphangiographic imaging.

#### 4.3.3 Cluster 3: The volume measurement of lymphedema

**Figure 11:**
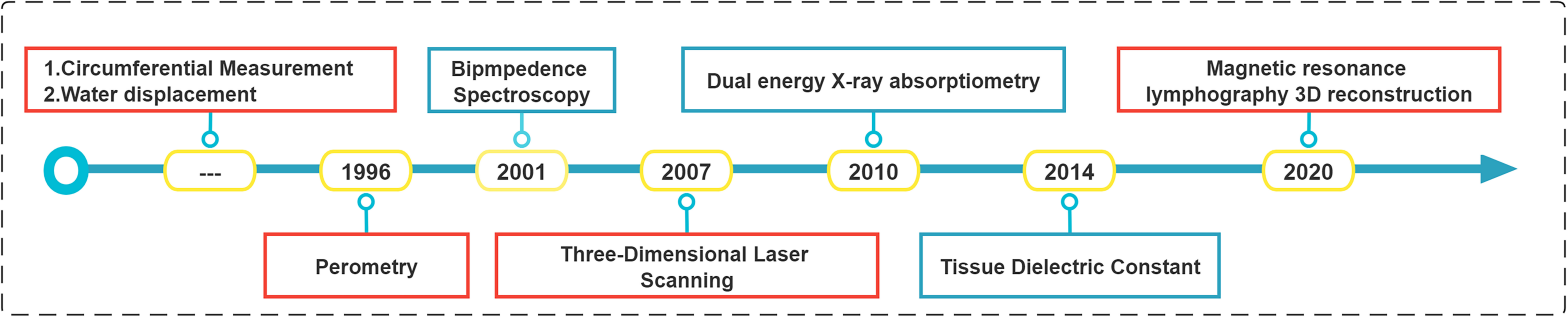
The historical progress of the volume measurement of lymphedema.

Currently, medical history collection and clinical examination (including circumferential measurements) are still the most common diagnostic modalities, and Cheng, Ming-Huei made a comprehensive staging by objectively evaluating circumferential measurements to guide the use of comprehensive decongestion therapy, lymphovascular venous anastomosis, and lymphatic grafting with vascular flaps in clinical practice (76). However, volumetric measurements are more responsive to the degree of limb swelling or treatment effect than circumferential dimensional measurements. The historical progress of the volume measurement of lymphedema is shown in **Figure 11**. Swelling is assessed by volumetric measurements, and historically, circumferential dimensional measurements have been used to calculate the assessment method (77–79). The water replacement method (80) was the most classic method. Later, infrared photoelectric measurements were gradually adopted (81), laser scanning 3D reconstruction measurement (82–84), and CT 3D reconstruction assessment (77). In the latest study, Cellina, Michaela et al. used ITK-SNAP to perform 3-dimensional reconstruction of MRL images of patients with lymphedema for effective assessment of limb volume. This method can kill two birds with one stone. On the one hand, we can use the advantage of MRL imaging to evaluate the structure and function of lymphatic vessels; on the other hand, we can achieve the purpose of evaluating the volume of limbs, so that we can make a better judgment of the disease before and after an operation. However, the clinical application remains to be developed and verified by clinical data (65).

In recent years, research on the early diagnosis of lymphedema has also become the research hotspot. Among them, the top word frequency is “Bioimpedance”, n=94. In the historical distribution of the top ten keywords, bioimpedance spectroscopy has become the fastest growing keyword in the past 20 years, with a 12-fold increase from 2000 to 2021; especially the number of papers after 2014 has increased significantly; and there are more contributions from Australian scholars, and these scholars show a strong trend of co-authorship. Bioimpedance spectroscopy can effectively determine whether the edematous limb is predominantly lymphoid or fatty in terms of tissue composition, thus avoiding the interference of weight changes in the determination of the disease, but effectively assessing the degree of limb edema and thus diagnosing lymphoedema earlier (85). In this way, the degree of limb edema can be effectively assessed without the interference of weight changes, leading to an earlier diagnosis of lymphedema. Over the past 20 years, bioimpedance spectroscopy has evolved from a proven technique to a valuable tool for screening, detection and monitoring of lymphedema, particularly in the upper extremities (21). Bioimpedance spectroscopy has been used repeatedly in prospective screening programs to initiate early treatment with the goal of reducing morbidity. His application in the lower extremity needs further validation. Another study buzzword is: “tissue dielectric constant (TDC)”, and the use of TDC measurements to quantify local tissue water content may help distinguish between untreated lymphedema and lipoedema in women with chronic leg swelling (86). Tissue dielectric constant measurements are sensitive, noninvasive, and applicable to any site, and are thought to be widely used in the future for early diagnosis and outcome follow-up of subclinical lymphedema.

#### 4.3.4 Cluster 4 and 5: Treatment and prognosis of lymphedema

**Figure 12:**
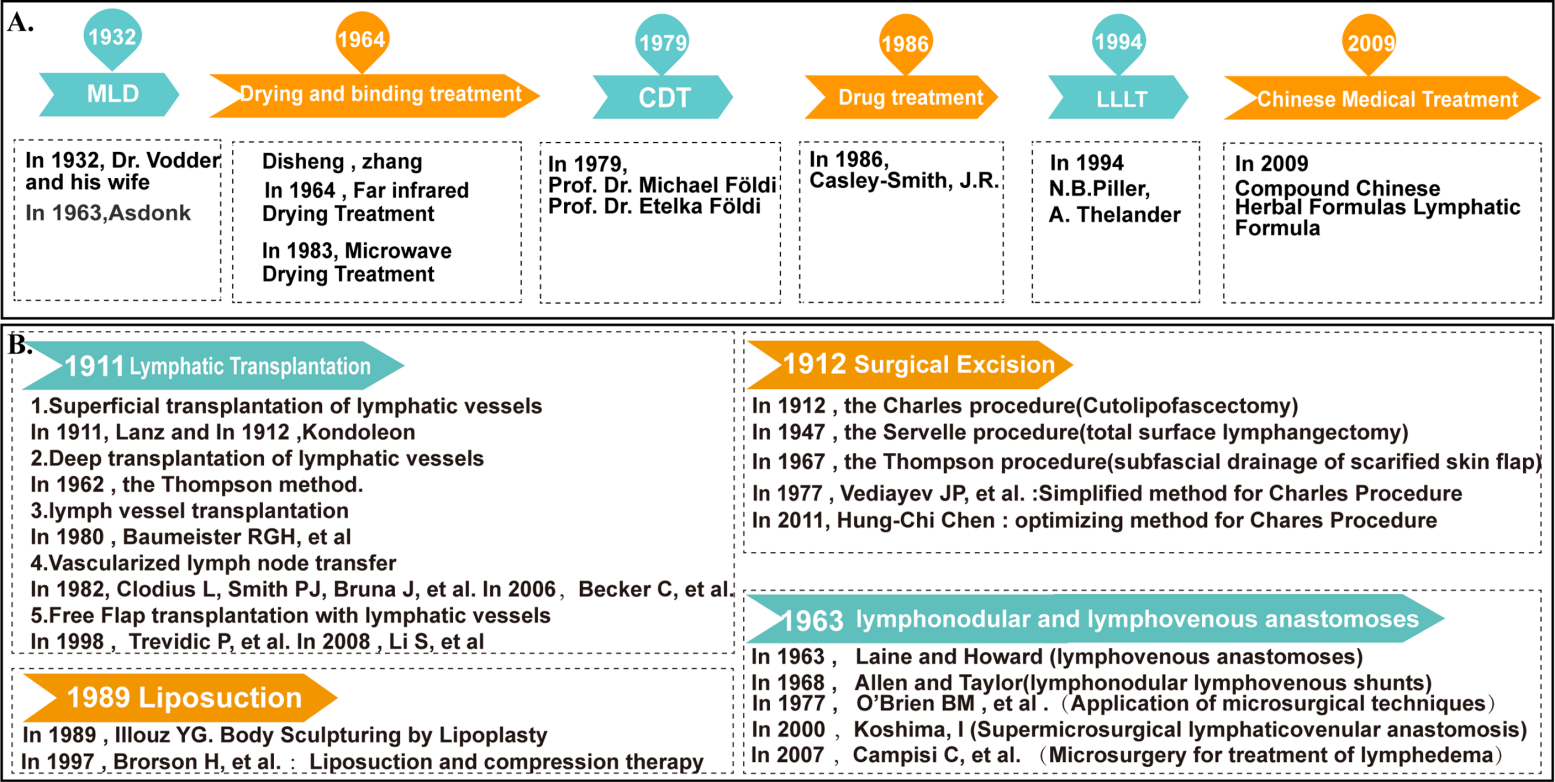
The development history of surgical and non-operative treatment of lymphedema. **(A)** The development history of non-operative treatment of lymphedema. **(B)** The development history of surgical treatment of lymphedema.

Treatment of lymphedema is performed with a combination of surgical and non-surgical treatments. The development of non-surgical treatment has historically included (MLD), drying and binding therapy, complete decongestion therapy (CDT), pneumatic compression therapy, pharmacotherapy, low-level laser therapy (LLLT), Chinese medical treatment, etc., as shown in **Figure 12**, but currently, the combined treatment model based on comprehensive decongestion therapy is still in effect (87–97). However, at present, the combined treatment model is based on comprehensive congestion therapy. Vascularized lymph node transfer and lymphovenous anastomosis remain the most common microsurgical techniques associated with the surgical management of this disease (13, 17). Other surgical treatment modalities include (lymphatic reconstruction surgery, surgical volume reduction surgery, Liposuction, etc.) (14–16, 35, 98–100), specific developments and additions are shown in **Figure 12**.

No single treatment modality can completely cure lymphedema, whether it is a non-surgical treatment, including diet, patient education, and patient self-management in conjunction with surgical treatment. Therefore, we pay more attention to the quality of life of patients with lymphedema. Health-related quality of life is a patient self-reported prognostic indicator that is commonly used to measure treatment outcome and is an independent predictor of patient survival. Cheng et al. showed that improvements in Health-related quality of life indicators can be recognized early and seem to correlate well with improvements in limb circumference measurements in the first year after surgery (76). In general, health-related quality-of-life measures can be used to assess the expected development and treatment of lymphedema in the head and neck, upper and lower extremities (101–105).

In summary, patients with suspected lymphedema can be screened out by subjective symptoms, medical history and physical examination. Early diagnosis of lymphedema can be done by tissue dielectric constant and bioimpedance spectroscopy; definite diagnosis of lymphedema can be done by MRL, ICG lymphadenography, and Lymphscintigraphy. However, there is no standardized diagnostic procedure. In the imaging of lymphatic vessels, more traditional imaging methods will also be used in combination with other examinations to improve the accuracy and reliability of the examination. The expectation is that using multiple methods seems more likely to be the gold standard for diagnosing lymphedema than using any one method. We know that accurate diagnosis and staging is essential to select the best treatment plan, based on the most widely accepted staging system that has been in place since 1985, the World Health Organization lymphedema staging (106, 107). Throughout history, the development of critical staging has remained only at the stage of clinical symptoms, and we have not been able to do a better staging of lymphedema at the pathological and anatomical levels to guide treatment. The existing ICG lymphography-based staging, Cheng’s staging and the latest Taiwan lymphography staging may be more definite in guiding clinical work. However, it is not yet widely used in clinical practice, and we look forward to exploring better staging with better diagnostic tools for guiding clinical practice and serving patients. In addition, with the level of lymphovascular imaging and the development of microsurgery, surgical interventions will play a greater role in the field of lymphedema. Stem cell therapy and lymphokine therapy to promote lymphatic vessel production are just some of the potential future treatment techniques. There are no uniformly effective treatment options for lymphedema and further exploration is needed. We may use physical therapy as the basis of lymphedema treatment, strive for early detection, early diagnosis, and early prevention; strictly based on the standardized staging and grading system, and adopt a multimodal combination of treatment under microsurgical conditions to personalize the treatment for patients so that they can obtain maximum benefit. Moreover, ARM and IRL are more promising options for reducing the incidence of lymphedema in patients with cancers involving lymph node therapy.

In fact, these keywords have a wide range; they can be interpreted as themes that reveal the mechanism, etiology, diagnosis, and treatment of lymphedema. Highly cited papers also showed great research value for contributing to the in-depth understanding of a field. Through our analysis, we found that the top 100 cited papers were consistent in terms of the themes of keywords, thereby showing the timelessness and importance of topics in the study of breast cancer-related lymphedema and lymphangiogenesis. On the whole, the study of lymphangiogenesis concerning the mechanism and treatment of lymphedema and breast cancer-related lymphedema is still the core topic of research.

## 5 Advantages and limitations

The advantage of our research are as follows: We conducted a comprehensive survey of the article in the field of lymphedema over the past 120 years, providing a quantitative and qualitative analysis of research results and quality across countries, institutions, and authors; We also constructed and visualized bibliometric networks through co-authorship and co-occurrence analysis; As far as we know, this is the first bibliometric analysis of the research trend of lymphedema; The results of the study enrich the knowledge base of Lymphedema and may help researchers to analyze the characteristics of research outputs, distribution of contributions, and knowledge mapping in the field of Lymphedema, thus seeking potential research topics and collaborators, and possibly encouraging further practice in this area. Nevertheless, our analysis has some limitations. One is that it is based on the WOS database rather than other databases such as PubMed, Scopus and GoogleScholar. Second, we did not specify bibliometric indicators such as H-index and impact factor; however, the qualitative and quantitative studies are fully descriptive of the topic we studied. Third, we seem to include only WOS’s English studies, but there are fewer publications in other languages, which reduces the interference with the results to a certain extent. Finally, as this is a developing field of research, we may have underestimated the contribution of different analyses of recently published studies because of their low frequency of citation, although some studies are published in high quality journals.

## 6 Conclusion

In conclusion, lymphedema is a growing topic in the article, and more and more scholars are devoted to the research of cancer-related lymphedema. With this study, we analyzed the characteristics of the output of lymphedema research, providing a historical perspective. It can be predicted that the research on the mechanism and treatment of Lymphedema and the prevention and treatment of BCRL will still be the core topics of future research; the progress of Lymphatic-vessel imaging and the development of lymphatic microsurgery will further play a role in the clinical work of Lymphedema. Meanwhile, the United States has been dominating this field for some time now and is likely to remain so for some time. Nevertheless, the intensity of research cooperation needs to be increased, especially in developing countries. In addition, these well-developed teams in lymphedema research such as Rockson, Stanley G’s team; Ward, Leigh C’s team; Devoogdt, Nele’s team; Partsch, Hugo’s team; Witte, MH’s team; Taghian, Alphonse G’s team; Chang David W’s team; Armer, Jane’s team; Koshima, Isao’s team, etc. are academic research groups that we can learn from and seek help and cooperation with in the long run. This study can potentially be instructive and can contribute to further research in the field of Lymphedema.

## Data availability statement

The raw data supporting the conclusions of this article will be made available by the authors, without undue reservation.

## Author contributions

Js-D, Dm-H, and Yd-Z designed the study. Yd-Z conducted the literature search. XZ, Xy-W, Yd-Z analyzed the data and wrote the paper. Js-D, Dm-H approved the final manuscript. All authors contributed to the article and approved the submitted version.

## Funding

This work was supported by the Key Laboratory Construction Project of Jilin Province (No. 21ZY17) and Jilin Provincial Health Science and Technology capacity Enhancement Project (No. 2021LC208).

## Conflict of interest

The authors declare that the research was conducted in the absence of any commercial or financial relationships that could be construed as a potential conflict of interest.

## Publisher’s note

All claims expressed in this article are solely those of the authors and do not necessarily represent those of their affiliated organizations, or those of the publisher, the editors and the reviewers. Any product that may be evaluated in this article, or claim that may be made by its manufacturer, is not guaranteed or endorsed by the publisher.

## Footnotes

1
https://clarivate.com/webofsciencegroup/support/wos/wos-core-collection/.
