# Supplementary material for: Visual analysis of global research output of lymphedema based on bibliometrics
Source: Front Oncol. 2022 Aug 5;12:926237. doi: 10.3389/fonc.2022.926237 (PMC9389543; doi:10.3389/fonc.2022.926237)
Supplement: Supplementary file 1 [file DataSheet_1.docx]

Supplementary Material

**Supplementary Table S1 |** The 23 valuable journals of lymphedema research

| **Name** | **Web of Science Documents** | **Times Cited** | **JIF Quartile** | **WoS Categories** | **Journal Impact Factor** | **Journal Normalized Citation Impact** |
| --- | --- | --- | --- | --- | --- | --- |
| **LYMPHOLOGY** | **551** | **8443** | **Q4** | **IMMUNOLOGY;PHYSIOLOGY** | **1.286** | **1.272** |
| **LYMPHATIC RESEARCH AND BIOLOGY** | **389** | **2815** | **Q3** | **MEDICINE, RESEARCH & EXPERIMENTAL;PHYSIOLOGY** | **2.589** | **0.944** |
| **PLASTIC AND RECONSTRUCTIVE SURGERY** | **250** | **6285** | **Q1** | **SURGERY** | **4.73** | **1.676** |
| **ANNALS OF SURGICAL ONCOLOGY** | **177** | **4178** | **Q1** | **ONCOLOGY;SURGERY** | **5.344** | **0.799** |
| **JOURNAL OF SURGICAL ONCOLOGY** | **136** | **2263** | **Q2** | **ONCOLOGY;SURGERY** | **3.454** | **1.559** |
| **ANNALS OF PLASTIC SURGERY** | **132** | **2541** | **Q4** | **SURGERY** | **1.539** | **1.620** |
| **MICROSURGERY** | **130** | **2104** | **Q2** | **SURGERY** | **2.425** | **1.695** |
| **SUPPORTIVE CARE IN CANCER** | **119** | **2201** | **Q1** | **HEALTH CARE SCIENCES & SERVICES;ONCOLOGY;REHABILITATION** | **3.603** | **1.118** |
| **BREAST CANCER RESEARCH AND TREATMENT** | **118** | **3309** | **Q2** | **ONCOLOGY** | **4.872** | **1.005** |
| **JOURNAL OF PLASTIC RECONSTRUCTIVE AND AESTHETIC SURGERY** | **104** | **1029** | **Q2** | **SURGERY** | **2.74** | **1.396** |
| **INTERNATIONAL JOURNAL OF RADIATION ONCOLOGY BIOLOGY PHYSICS** | **103** | **2054** | **Q1** | **ONCOLOGY;RADIOLOGY, NUCLEAR MEDICINE & MEDICAL IMAGING** | **7.038** | **0.678** |
| **JOURNAL OF CLINICAL ONCOLOGY** | **93** | **5110** | **Q1** | **ONCOLOGY** | **44.544** | **0.600** |
| **CANCER** | **89** | **6934** | **Q1** | **ONCOLOGY** | **6.86** | **1.344** |
| **GYNECOLOGIC ONCOLOGY** | **83** | **2525** | **Q1** | **OBSTETRICS & GYNECOLOGY;ONCOLOGY** | **5.482** | **1.292** |
| **INTERNATIONAL JOURNAL OF GYNECOLOGICAL CANCER** | **80** | **1444** | **Q2** | **OBSTETRICS & GYNECOLOGY;ONCOLOGY** | **3.437** | **1.263** |
| **JOURNAL OF RECONSTRUCTIVE MICROSURGERY** | **68** | **1456** | **Q2** | **SURGERY** | **2.873** | **1.755** |
| **ONCOLOGY NURSING FORUM** | **65** | **703** | **Q2** | **NURSING;ONCOLOGY** | **2.172** | **0.597** |
| **PLOS ONE** | **63** | **1263** | **Q2** | **BIOLOGY;MULTIDISCIPLINARY SCIENCES** | **3.24** | **0.808** |
| **CANCER RESEARCH** | **63** | **570** | **Q1** | **ONCOLOGY** | **12.701** | **0.171** |
| **JOURNAL OF THE AMERICAN ACADEMY OF DERMATOLOGY** | **60** | **1567** | **Q1** | **DERMATOLOGY** | **11.527** | **0.755** |
| **PHYSICAL THERAPY** | **50** | **1403** | **n/a** | **ORTHOPEDICS;REHABILITATION** | **n/a** | **1.021** |
| **ACTA ONCOLOGICA** | **39** | **1609** | **Q3** | **ONCOLOGY** | **4.089** | **1.803** |
| **AMERICAN JOURNAL OF HUMAN GENETICS** | **27** | **2206** | **Q1** | **GENETICS & HEREDITY** | **11.025** | **0.784** |

**Supplementary Table S2 |** The top 100 highly cited publications.

| **Rank** | **Title** | **Authors** | **Jouanal** | **Publication Year** | **Total Citations** | **Average Per Year** |
| --- | --- | --- | --- | --- | --- | --- |
| **1** | Lymphatic Mapping And Sentinel Lymphadenectomy For Breast-Cancer | Giuliano, Ae; Kirgan, Dm; Guenther, Jm; Morton, Dl | Annals Of Surgery | 1994 | 2007 | 71.68 |
| **2** | Endothelial/Pericyte Interactions | Armulik, A; Abramsson, A; Betsholtz, C | Circulation Research | 2005 | 1321 | 77.71 |
| **3** | Molecular Regulation Of Angiogenesis And Lymphangiogenesis | Adams, Ralf H.; Alitalo, Kari | Nature Reviews Molecular Cell Biology | 2007 | 1262 | 84.13 |
| **4** | Randomized Multicenter Trial Of Sentinel Node Biopsy Versus Standard Axillary Treatment In Operable Breast Cancer: The Almanac Trial | Mansel, Re; Fallowfield, L; Kissin, M; Goyal, A; Newcombe, Rg; Dixon, Jm; Yiangou, C; Horgan, K; Bundred, N; Monypenny, I; England, D; Sibbering, M; Abdullah, Tj; Barr, L; Chetty, U; Sinnett, Dh; Fleissig, A; Clarke, D; Ell, Pj | Journal Of The National Cancer Institute | 2006 | 1057 | 66.06 |
| **5** | Vascular Endothelial Growth Factor C Is Required For Sprouting Of The First Lymphatic Vessels From Embryonic Veins | Karkkainen, Mj; Haiko, P; Sainio, K; Partanen, J; Taipale, J; Petrova, Tv; Jeltsch, M; Jackson, Dg; Talikka, M; Rauvala, H; Betsholtz, C; Alitalo, K | Nature Immunology | 2004 | 917 | 50.94 |
| **6** | Lymphangiogenesis In Development And Human Disease | Alitalo, K; Tammela, T; Petrova, Tv | Nature | 2005 | 864 | 50.82 |
| **7** | Signal Transduction By Vegf Receptors In Regulation Of Angiogenesis And Lymphangiogenesis | Shibuya, M; Claesson-Welsh, L | Experimental Cell Research | 2006 | 735 | 45.94 |
| **8** | Long-Range Control Of Gene Expression: Emerging Mechanisms And Disruption In Disease | Kleinjan, Da; Van Heyningen, V | American Journal Of Human Genetics | 2005 | 639 | 37.59 |
| **9** | Effects Of Aerobic And Resistance Exercise In Breast Cancer Patients Receiving Adjuvant Chemotherapy: A Multicenter Randomized Controlled Trial | Courneya, Kerry S.; Segal, Roanne J.; Mackey, John R.; Gelmon, Karen; Reid, Robert D.; Friedenreich, Christine M.; Ladha, Aliya B.; Proulx, Caroline; Vallance, Jeffrey K. H.; Lane, Kirstin; Yasui, Yutaka; Mckenzie, Donald C. | Journal Of Clinical Oncology | 2007 | 638 | 42.53 |
| **10** | Regional Nodal Irradiation In Early-Stage Breast Cancer | Whelan, Timothy J.; Olivotto, Ivo A.; Parulekar, Wendy R.; Ackerman, Ida; Chua, Boon H.; Nabid, Abdenour; Vallis, Katherine A.; White, Julia R.; Rousseau, Pierre; Fortin, Andre; Pierce, Lori J.; Manchul, Lee; Chafe, Susan; Nolan, Maureen C.; Craighead, Peter; Bowen, Julie; Mccready, David R.; Pritchard, Kathleen I.; Gelmon, Karen; Murray, Yvonne; Chapman, Judy-Anne W.; Chen, Bingshu E.; Levine, Mark N. | New England Journal Of Medicine | 2015 | 607 | 86.71 |
| **11** | Lymphangiosarcoma In Postmastectomy Lymphedema - A Report Of 6 Cases In Elephantiasis Chirurgica | Stewart, Fw; Treves, N | Cancer | 1948 | 597 | 8.07 |
| **12** | Completion Dissection Or Observation For Sentinel-Node Metastasis In Melanoma | Faries, M. B.; Thompson, J. F.; Cochran, A. J.; Andtbacka, R. H.; Mozzillo, N.; Zager, J. S.; Jahkola, T.; Bowles, T. L.; Testori, A.; Beitsch, P. D.; Hoekstra, H. J.; Moncrieff, M.; Ingvar, C.; Wouters, M. W. J. M.; Sabel, M. S.; Levine, E. A.; Agnese, D.; Henderson, M.; Dummer, R.; Rossi, C. R.; Neves, R. I.; Trocha, S. D.; Wright, F.; Byrd, D. R.; Matter, M.; Hsueh, E.; Mackenzie-Ross, A.; Johnson, D. B.; Terheyden, P.; Berger, A. C.; Huston, T. L.; Wayne, J. D.; Smithers, B. M.; Neuman, H. B.; Schneebaum, S.; Gershenwald, J. E.; Ariyan, C. E.; Desai, D. C.; Jacobs, L.; Mcmasters, K. M.; Gesierich, A.; Hersey, P.; Bines, S. D.; Kane, J. M.; Barth, R. J.; Mckinnon, G.; Farma, J. M.; Schultz, E.; Vidal-Sicart, S.; Hoefer, R. A.; Lewis, J. M.; Scheri, R.; Kelley, M. C.; Nieweg, O. E.; Noyes, R. D.; Hoon, D. S. B.; Wang, H. -J.; Elashoff, D. A.; Elashoff, R. M. | New England Journal Of Medicine | 2017 | 596 | 119.2 |
| **13** | Inhibition Of Lymphangiogenesis With Resulting Lymphedema In Transgenic Mice Expressing Soluble Vegf Receptor-3 | Makinen, T; Jussila, L; Veikkola, T; Karpanen, T; Kettunen, Mi; Pulkkanen, Kj; Kauppinen, R; Jackson, Dg; Kubo, H; Nishikawa, Si; Yla-Herttuala, S; Alitalo, K | Nature Medicine | 2001 | 566 | 26.95 |
| **14** | The Lymphatic Vasculature In Disease | Alitalo, Kari | Nature Medicine | 2011 | 555 | 50.45 |
| **15** | Molecular Mechanisms Of Lymphangiogenesis In Health And Disease | Alitalo, K; Carmeliet, P | Cancer Cell | 2002 | 509 | 25.45 |
| **16** | Surgical Complications Associated With Sentinel Lymph Node Dissection (Slnd) Plus Axillary Lymph Node Dissection Compared With Slnd Alone In The American College Of Surgeons Oncology Group Trial Z0011 | Lucci, Anthony; Mccall, Linda Mackie; Beitsch, Peter D.; Whitworth, Patrick W.; Reintgen, Douglas S.; Blumencranz, Peter W.; Leitch, A. Marilyn; Saha, Sukumal; Hunt, Kelly K.; Giuliano, Armando E. | Journal Of Clinical Oncology | 2007 | 508 | 33.87 |
| **17** | Risk Of Lymphedema Following The Treatment Of Breast-Cancer | Kissin, Mw; Dellarovere, Gq; Easton, D; Westbury, G | British Journal Of Surgery | 1986 | 498 | 13.83 |
| **18** | T1 Alpha/Podoplanin Deficiency Disrupts Normal Lymphatic Vasculature Formation And Causes Lymphedema | Schacht, V; Ramirez, Mi; Hong, Yk; Hirakawa, S; Feng, D; Harvey, N; Williams, M; Dvorak, Am; Dvorak, Hf; Oliver, G; Detmar, M | Embo Journal | 2003 | 493 | 25.95 |
| **19** | Signal Transduction By Vascular Endothelial Growth Factor Receptors | Koch, Sina; Claesson-Welsh, Lena | Cold Spring Harbor Perspectives In Medicine | 2012 | 483 | 48.3 |
| **20** | Lymphatic Endothelial Reprogramming Of Vascular Endothelial Cells By The Prox-1 Homeobox Transcription Factor | Petrova, Tv; Makinen, T; Makela, Tp; Saarela, J; Virtanen, I; Ferrell, Re; Finegold, Dn; Kerjaschki, D; Yla-Herttuala, S; Alitalo, K | Embo Journal | 2002 | 482 | 24.1 |
| **21** | Up-Regulation Of The Lymphatic Marker Podoplanin, A Mucin-Type Transmembrane Glycoprotein, In Human Squamous Cell Carcinomas And Germ Cell Tumors | Schacht, V; Dadras, Ss; Johnson, La; Jackson, Dg; Hong, Yk; Detmar, M | American Journal Of Pathology | 2005 | 474 | 27.88 |
| **22** | Sentinel Node Dissection Is Safe In The Treatment Of Early-Stage Vulvar Cancer | Van Der Zee, Ate G. J.; Oonk, Maaike H.; De Hullu, Joanne A.; Ansink, Anca C.; Vergote, Ignace; Verheijen, Rene H.; Maggioni, Angelo; Gaarenstroom, Katja N.; Baldwin, Peter J.; Van Dorst, Eleonore B.; Van Der Velden, Jacobus; Hermans, Ralph H.; Van Der Putten, Hans; Drouin, Pierre; Schneider, Achim; Sluiter, Wim J. | Journal Of Clinical Oncology | 2008 | 471 | 33.64 |
| **23** | Pathogenesis Of Persistent Lymphatic Vessel Hyperplasia In Chronic Airway Inflammation | Baluk, P; Tammela, T; Ator, E; Lyubynska, N; Achen, Mg; Hicklin, Dj; Jeltsch, M; Petrova, Tv; Pytowski, B; Stacker, Sa; Yla-Herttuala, S; Jackson, Dg; Alitalo, K; Mcdonald, Dm | Journal Of Clinical Investigation | 2005 | 448 | 26.35 |
| **24** | Missense Mutations Interfere With Vegfr-3 Signalling In Primary Lymphoedema | Karkkainen, Mj; Ferrell, Re; Lawrence, Ec; Kimak, Ma; Levinson, Kl; Mctigue, Ma; Alitalo, K; Finegold, Dn | Nature Genetics | 2000 | 442 | 20.09 |
| **25** | Lymphedema In A Cohort Of Breast Carcinoma Survivors 20 Years After Diagnosis | Petrek, Ja; Senie, Rt; Peters, M; Rosen, Pp | Cancer | 2001 | 436 | 20.76 |
| **26** | Mutations In Foxc2 (Mfh-1), A Forkhead Family Transcription Factor, Are Responsible For The Hereditary Lymphedema-Distichiasis Syndrome | Fang, Jm; Dagenais, Sl; Erickson, Rp; Arlt, Mf; Glynn, Mw; Gorski, Jl; Seaver, Lh; Glover, Tw | American Journal Of Human Genetics | 2000 | 412 | 18.73 |
| **27** | A Model For Gene Therapy Of Human Hereditary Lymphedema | Karkkainen, Mj; Saaristo, A; Jussila, L; Karila, Ka; Lawrence, Ec; Pajusola, K; Bueler, H; Eichmann, A; Kauppinen, R; Kettunen, Mi; Yla-Herttuala, S; Finegold, Dn; Ferrell, Re; Alitalo, K | Proceedings Of The National Academy Of Sciences Of The United States Of America | 2001 | 411 | 19.57 |
| **28** | Pericytes. Morphofunction, Interactions And Pathology In A Quiescent And Activated Mesenchymal Cell Niche | Diaz-Flores, L.; Gutierrez, R.; Madrid, J. F.; Varela, H.; Valladares, F.; Acosta, E.; Martin-Vasallo, P.; Diaz-Flores, L., Jr. | Histology And Histopathology | 2009 | 407 | 31.31 |
| **29** | The Diagnosis And Treatment Of Peripheral Lymphedema: 2013 Consensus Document Of The International Society Of Lymphology | [Anonymous] | Lymphology | 2013 | 406 | 45.11 |
| **30** | Defective Valves And Abnormal Mural Cell Recruitment Underlie Lymphatic Vascular Failure In Lymphedema Distichiasis | Petrova, Tv; Karpanen, T; Norrmen, C; Mellor, R; Tamakoshi, T; Finegold, D; Ferrell, R; Kerjaschki, D; Mortimer, P; Yla-Herttuala, S; Miura, N; Alitalo, K | Nature Medicine | 2004 | 401 | 22.28 |
| **31** | Morbidity Following Sentinel Lymph Node Biopsy Versus Axillary Lymph Node Dissection For Patients With Breast Carcinoma | Schrenk, P; Rieger, R; Shamiyeh, A; Wayand, W | Cancer | 2000 | 398 | 18.09 |
| **32** | Prevalence Of Lymphedema In Women With Breast Cancer 5 Years After Sentinel Lymph Node Biopsy Or Axillary Dissection: Objective Measurements | Mclaughlin, Sarah A.; Wright, Mary J.; Morris, Katherine T.; Giron, Gladys L.; Sampson, Michelle R.; Brockway, Julia P.; Hurley, Karen E.; Riedel, Elyn R.; Van Zee, Kimberly J. | Journal Of Clinical Oncology | 2008 | 387 | 27.64 |
| **33** | Surgical Complications Associated With Sentinel Lymph Node Biopsy: Results From A Prospective International Cooperative Group Trial | Wilke, Lg; Mccall, Lm; Posther, Ke; Whitworth, Pw; Reintgen, Ds; Leitch, Am; Gabram, Sga; Lucci, A; Cox, Ce; Hunt, Kk; Herndon, Je; Giuliano, Ae | Annals Of Surgical Oncology | 2006 | 386 | 24.13 |
| **34** | Human Fox Gene Family (Review) | Katoh, M; Katoh, M | International Journal Of Oncology | 2004 | 379 | 21.06 |
| **35** | Arm Edema In Breast Cancer Patients | Erickson, Vs; Pearson, Ml; Ganz, Pa; Adams, J; Kahn, Kl | Journal Of The National Cancer Institute | 2001 | 363 | 17.29 |
| **36** | Gata2 Deficiency: A Protean Disorder Of Hematopoiesis, Lymphatics, And Immunity | Spinner, Michael A.; Sanchez, Lauren A.; Hsu, Amy P.; Shaw, Pamela A.; Zerbe, Christa S.; Calvo, Katherine R.; Arthur, Diane C.; Gu, Wenjuan; Gould, Christine M.; Brewer, Carmen C.; Cowen, Edward W.; Freeman, Alexandra F.; Olivier, Kenneth N.; Uzel, Gulbu; Zelazny, Adrian M.; Daub, Janine R.; Spalding, Christine D.; Claypool, Reginald J.; Giri, Neelam K.; Alter, Blanche P.; Mace, Emily M.; Orange, Jordan S.; Cuellar-Rodriguez, Jennifer; Hickstein, Dennis D.; Holland, Steven M. | Blood | 2014 | 361 | 45.13 |
| **37** | Lymphatic Vascular Defects Promoted By Prox1 Haploinsufficiency Cause Adult-Onset Obesity | Harvey, Nl; Srinivasan, Rs; Dillard, Me; Johnson, Nc; Witte, Mh; Boyd, K; Sleeman, Mw; Oliver, G | Nature Genetics | 2005 | 355 | 20.88 |
| **38** | Weight Lifting In Women With Breast-Cancer-Related Lymphedema | Schmitz, Kathryn H.; Ahmed, Rehana L.; Troxel, Andrea; Cheville, Andrea; Smith, Rebecca; Lewis-Grant, Lorita; Bryan, Cathy J.; Williams-Smith, Catherine T.; Greene, Quincy P. | New England Journal Of Medicine | 2009 | 350 | 26.92 |
| **39** | Lymphedema - A Comprehensive Review | Warren, Anne G.; Brorson, Hakan; Borud, Loren J.; Slavin, Sumner A. | Annals Of Plastic Surgery | 2007 | 349 | 23.27 |
| **40** | Angiosarcoma Of Soft Tissue - A Study Of 80 Cases | Meis-Kindblom, Jm; Kindblom, Lg | American Journal Of Surgical Pathology | 1998 | 334 | 13.92 |
| **41** | Vascular Endothelial Growth Factor Receptors In The Regulation Of Angiogenesis And Lymphangiogenesis | Karkkainen, Mj; Petrova, Tv | Oncogene | 2000 | 333 | 15.14 |
| **42** | Lymphedema | Rockson, Sg | American Journal Of Medicine | 2001 | 319 | 15.19 |
| **43** | Lymphatic Vasculature: Development, Molecular Regulation And Role In Tumor Metastasis And Inflammation | Saharinen, P; Tammela, T; Karkkainen, Mj; Alitalo, K | Trends In Immunology | 2004 | 314 | 17.44 |
| **44** | Postmastectomy Lymphedema - Long-Term Results Following Microsurgical Lymph Node Transplantation | Becker, C; Assouad, J; Riquet, M; Hidden, G | Annals Of Surgery | 2006 | 313 | 19.56 |
| **45** | Incidence Of Breast Carcinoma-Related Lymphedema | Petrek, Ja; Heelan, Mc | Cancer | 1998 | 312 | 13 |
| **46** | Mutations In Gata2 Cause Primary Lymphedema Associated With A Predisposition To Acute Myeloid Leukemia (Emberger Syndrome) | Ostergaard, Pia; Simpson, Michael A.; Connell, Fiona C.; Steward, Colin G.; Brice, Glen; Woollard, Wesley J.; Dafou, Dimitra; Kilo, Tatjana; Smithson, Sarah; Lunt, Peter; Murday, Victoria A.; Hodgson, Shirley; Keenan, Russell; Pilz, Daniela T.; Martinez-Corral, Ines; Makinen, Taija; Mortimer, Peter S.; Jeffery, Steve; Trembath, Richard C.; Mansour, Sahar | Nature Genetics | 2011 | 310 | 28.18 |
| **47** | Preoperative Assessment Enables The Early Diagnosis And Successful Treatment Of Lymphedema | Gergich, Nicole L. Stout; Pfalzer, Lucinda A.; Mcgarvey, Charles; Springer, Barbara; Gerber, Lynn H.; Soballe, Peter | Cancer | 2008 | 300 | 21.43 |
| **48** | Vascular Growth Factors And Lymphangiogenesis | Jussila, L; Alitalo, K | Physiological Reviews | 2002 | 290 | 14.5 |
| **49** | Incidence, Treatment Costs, And Complications Of Lymphedema After Breast Cancer Among Women Of Working Age: A 2-Year Follow-Up Study | Shih, Ya-Chen Tina; Xu, Ying; Cormier, Janice N.; Giordano, Sharon; Ridner, Sheila H.; Buchholz, Thomas A.; Perkins, George H.; Elting, Linda S. | Journal Of Clinical Oncology | 2009 | 289 | 22.23 |
| **50** | Lymphatic Vasculature Development | Oliver, G | Nature Reviews Immunology | 2004 | 281 | 15.61 |
| **51** | Congenital Hereditary Lymphedema Caused By A Mutation That Inactivates Vegfr3 Tyrosine Kinase | Irrthum, A; Karkkainen, Mj; Devriendt, K; Alitalo, K; Vikkula, M | American Journal Of Human Genetics | 2000 | 274 | 12.45 |
| **52** | Mutations In The Transcription Factor Gene Sox18 Underlie Recessive And Dominant Forms Of Hypotrichosis-Lymphedema-Telangiectasia | Irrthum, A; Devriendt, K; Chitayat, D; Matthijs, G; Glade, C; Steijlen, Pm; Fryns, Jp; Van Steensel, Mam; Vikkula, M | American Journal Of Human Genetics | 2003 | 273 | 14.37 |
| **53** | Lymphedema After Breast Cancer: Incidence, Risk Factors, And Effect On Upper Body Function | Hayes, Sandra C.; Janda, Monika; Cornish, Bruce; Battistutta, Diana; Newman, Beth | Journal Of Clinical Oncology | 2008 | 273 | 19.5 |
| **54** | The Third Circulation: Radionuclide Lymphoscintigraphy In The Evaluation Of Lymphedema | Szuba, A; Shin, Ws; Strauss, Hw; Rockson, S | Journal Of Nuclear Medicine | 2003 | 273 | 14.37 |
| **55** | Obesity And The Skin: Skin Physiology And Skin Manifestations Of Obesity | Yosipovitch, Gil; Devore, Amy; Dawn, Aerlyn | Journal Of The American Academy Of Dermatology | 2007 | 262 | 17.47 |
| **56** | The Rediscovery Of The Lymphatic System: Old And New Insights Into The Development And Biological Function Of The Lymphatic Vasculature | Oliver, G; Detmar, M | Genes & Development | 2002 | 261 | 13.05 |
| **57** | The Incidence Of Symptomatic Lower-Extremity Lymphedema Following Treatment Of Uterine Corpus Malignancies: A 12-Year Experience At Memorial Sloan-Kettering Cancer Center | Abu-Rustum, Nadeem R.; Alektiar, Kaled; Iasonos, Alexia; Lev, Gali; Sonoda, Yukio; Aghajanian, Carol; Chi, Dennis S.; Barakat, Richard R. | Gynecologic Oncology | 2006 | 257 | 16.06 |
| **58** | Alternatively Spliced Vascular Endothelial Growth Factor Receptor-2 Is An Essential Endogenous Inhibitor Of Lymphatic Vessel Growth | Albuquerque, Romulo J. C.; Hayashi, Takahiko; Cho, Won Gil; Kleinman, Mark E.; Dridi, Sami; Takeda, Atsunobu; Baffi, Judit Z.; Yamada, Kiyoshi; Kaneko, Hiroki; Green, Martha G.; Chappell, Joe; Wilting, Joerg; Weich, Herbert A.; Yamagami, Satoru; Amano, Shiro; Mizuki, Nobuhisa; Alexander, Jonathan S.; Peterson, Martha L.; Brekken, Rolf A.; Hirashima, Masanori; Capoor, Seema; Usui, Tomohiko; Ambati, Balamurali K.; Ambati, Jayakrishna | Nature Medicine | 2009 | 251 | 19.31 |
| **59** | Molecular Biology And Pathology Of Lymphangiogenesis | Karpanen, Terhi; Alitalo, Karl | Annual Review Of Pathology-Mechanisms Of Disease | 2008 | 250 | 17.86 |
| **60** | Weight Lifting For Women At Risk For Breast Cancer-Related Lymphedema A Randomized Trial | Schmitz, Kathryn H.; Ahmed, Rehana L.; Troxel, Andrea B.; Cheville, Andrea; Lewis-Grant, Lorita; Smith, Rebecca; Bryan, Cathy J.; Williams-Smith, Catherine T.; Chittams, Jesse | Jama-Journal Of The American Medical Association | 2010 | 248 | 20.67 |
| **61** | Lymphedema In Breast Cancer Survivors: Incidence, Degree, Time Course, Treatment, And Symptoms | Norman, Sandra A.; Localio, A. Russell; Potashnik, Sheryl L.; Torpey, Heather A. Simoes; Kallan, Michael J.; Weber, Anita L.; Miller, Linda T.; Demichele, Angela; Solin, Lawrence J. | Journal Of Clinical Oncology | 2009 | 248 | 19.08 |
| **62** | Therapeutic Differentiation And Maturation Of Lymphatic Vessels After Lymph Node Dissection And Transplantation | Tammela, Tuomas; Saaristo, Anne; Holopainen, Tanja; Lyytikkae, Johannes; Kotronen, Anna; Pitkonen, Miia; Abo-Ramadan, Usama; Ylae-Herttuala, Seppo; Petrova, Tatiana V.; Alitalo, Kari | Nature Medicine | 2007 | 248 | 16.53 |
| **63** | The Psychological Morbidity Of Breast Cancer-Related Arm Swelling - Psychological Morbidity Of Lymphedema | Tobin, Mb; Lacey, Hj; Meyer, L; Mortimer, Ps | Cancer | 1993 | 245 | 8.45 |
| **64** | Lymphatic Vascular Morphogenesis In Development, Physiology, And Disease | Schulte-Merker, Stefan; Sabine, Amelie; Petrova, Tatiana V. | Journal Of Cell Biology | 2011 | 241 | 21.91 |
| **65** | Lymphedema And Quality Of Life In Breast Cancer Survivors: The Iowa Women'S Health Study | Ahmed, Rehana L.; Prizment, Anna; Lazovich, Deann; Schmitz, Kathryn H.; Folsom, Aaron R. | Journal Of Clinical Oncology | 2008 | 239 | 17.07 |
| **66** | Supermicrosurgical Lymphaticovenular Anastomosis For The Treatment Of Lymphedema In The Upper Extremities | Koshima, I; Inagawa, K; Urushibara, K; Moriguchi, T | Journal Of Reconstructive Microsurgery | 2000 | 239 | 10.86 |
| **67** | Chronic Arm Morbidity After Curative Breast Cancer Treatment: Prevalence And Impact On Quality Of Life | Kwan, W; Jackson, J; Weir, Lm; Dingee, C; Mcgregor, G; Olivotto, Ia | Journal Of Clinical Oncology | 2002 | 237 | 11.85 |
| **68** | Lymphedema After Gynecological Cancer Treatment - Prevalence, Correlates, And Supportive Care Needs | Beesley, Vanessa; Janda, Monika; Eakin, Elizabeth; Obermair, Andreas; Battistutta, Diana | Cancer | 2007 | 236 | 15.73 |
| **69** | Morbidity Of Sentinel Lymph Node Biopsy (Sln) Alone Versus Sln And Completion Axillary Lymph Node Dissection After Breast Cancer Surgery - A Prospective Swiss Multicenter Study On 659 Patients | Langer, Igor; Guller, Ulrich; Berclaz, Gilles; Koechli, Ossi R.; Schaer, Gabriel; Fehr, Mathias K.; Hess, Thomas; Oertli, Daniel; Bronz, Lucio; Schnarwyler, Beate; Wight, Edward; Uehlinger, Urs; Infanger, Eduard; Burger, Daniel; Zuber, Markus | Annals Of Surgery | 2007 | 235 | 15.67 |
| **70** | Lymphedema Beyond Breast Cancer | Cormier, Janice N.; Askew, Robert L.; Mungovan, Kristi S.; Xing, Yan; Ross, Merrick I.; Armer, Jane M. | Cancer | 2010 | 235 | 19.58 |
| **71** | Mechanotransduction, Prox1, And Foxc2 Cooperate To Control Connexin37 And Calcineurin During Lymphatic-Valve Formation | Sabine, Amelie; Agalarov, Yan; Maby-El Hajjami, Helene; Jaquet, Muriel; Haegerling, Rene; Pollmann, Cathrin; Bebber, Damien; Pfenniger, Anna; Miura, Naoyuki; Dormond, Olivier; Calmes, Jean-Marie; Adams, Ralf H.; Maekinen, Taija; Kiefer, Friedemann; Kwak, Brenda R.; Petrova, Tatiana V. | Developmental Cell | 2012 | 234 | 23.4 |
| **72** | The Global Programme To Eliminate Lymphatic Filariasis: Health Impact After 8 Years | Ottesen, Eric A.; Hooper, Pamela J.; Bradley, Mark; Biswas, Gautam | Plos Neglected Tropical Diseases | 2008 | 234 | 16.71 |
| **73** | Clinical Practice Guidelines On The Evidence-Based Use Of Integrative Therapies During And After Breast Cancer Treatment | Greenlee, Heather; Dupont-Reyes, Melissa J.; Balneaves, Lynda G.; Carlson, Linda E.; Cohen, Misha R.; Deng, Gary; Johnson, Jillian A.; Mumber, Matthew; Seely, Dugald; Zick, Suzanna M.; Boyce, Lindsay M.; Tripathy, Debu | Ca-A Cancer Journal For Clinicians | 2017 | 232 | 46.4 |
| **74** | A Prospective Analysis Of 100 Consecutive Lymphovenous Bypass Cases For Treatment Of Extremity Lymphedema | Chang, David W.; Suami, Hiroo; Skoracki, Roman | Plastic And Reconstructive Surgery | 2013 | 230 | 25.56 |
| **75** | Characteristic Indocyanine Green Lymphography Findings In Lower Extremity Lymphedema: The Generation Of A Novel Lymphedema Severity Staging System Using Dermal Backflow Patterns | Yamamoto, Takumi; Narushima, Mitsunaga; Doi, Kentaro; Oshima, Azusa; Ogata, Fusa; Mihara, Makoto; Koshima, Isao; Mundinger, Gerhard S. | Plastic And Reconstructive Surgery | 2011 | 229 | 20.82 |
| **76** | Integrin-Alpha 9 Is Required For Fibronectin Matrix Assembly During Lymphatic Valve Morphogenesis | Bazigou, Eleni; Xie, Sherry; Chen, Chun; Weston, Anne; Miura, Naoyuki; Sorokin, Lydia; Adams, Ralf; Muro, Andres F.; Sheppard, Dean; Makinen, Taija | Developmental Cell | 2009 | 226 | 17.38 |
| **77** | Early Diagnosis Of Lymphedema Using Multiple Frequency Bioimpedance | Cornish, Bh; Chapman, M; Hirst, C; Mirolo, B; Bunce, Ih; Ward, Lc; Thomas, Bj | Lymphology | 2001 | 224 | 10.67 |
| **78** | Foxc2 Controls Formation And Maturation Of Lymphatic Collecting Vessels Through Cooperation With Nfatc1 | Norrmen, Camilla; Ivanov, Konstantin I.; Cheng, Jianpin; Zangger, Nadine; Delorenzi, Mauro; Jaquet, Muriel; Miura, Naoyuki; Puolakkainen, Pauli; Horsley, Valerie; Hu, Junhao; Augustin, Hellmut G.; Ylae-Herttuala, Seppo; Alitalo, Kari; Petrova, Tatiana V. | Journal Of Cell Biology | 2009 | 220 | 16.92 |
| **79** | Vascularized Groin Lymph Node Transfer Using The Wrist As A Recipient Site For Management Of Postmastectomy Upper Extremity Lymphedema | Lin, Cheng-Hung; Ali, Rozina; Chen, Shin-Cheh; Wallace, Chris; Chang, Yu-Chen; Chen, Hung-Chi; Cheng, Ming-Huei | Plastic And Reconstructive Surgery | 2009 | 220 | 16.92 |
| **80** | Lymphedema: A Primer On The Identification And Management Of A Chronic Condition In Oncologic Treatment | Lawenda, Brian D.; Mondry, Tammy E.; Johnstone, Peter A. S. | Ca-A Cancer Journal For Clinicians | 2009 | 218 | 16.77 |
| **81** | Long-Term Adjustment Of Survivors Of Early-Stage Breast Carcinoma, 20 Years After Adjuvant Chemotherapy | Kornblith, Ab; Herndon, Je; Weiss, Rb; Zhang, Cf; Zuckerman, El; Rosenberg, S; Mertz, M; Payne, D; Massie, Mj; Holland, Jf; Wingate, P; Norton, L; Holland, Jc | Cancer | 2003 | 216 | 11.37 |
| **82** | Reliability And Validity Of Arm Volume Measurements For Assessment Of Lymphedema | Taylor, R; Jayasinghe, Uw; Koelmeyer, L; Ung, O; Boyages, J | Physical Therapy | 2006 | 215 | 13.44 |
| **83** | Incidence And Risk Of Arm Oedema Following Treatment For Breast Cancer: A Three-Year Follow-Up Study | Clark, B; Sitzia, J; Harlow, W | Qjm-An International Journal Of Medicine | 2005 | 214 | 12.59 |
| **84** | Exercise For Women Receiving Adjuvant Therapy For Breast Cancer | Markes, M.; Brockow, T.; Resch, K. L. | Cochrane Database Of Systematic Reviews | 2006 | 209 | 13.06 |
| **85** | Postoperative Complications After Vulvectomy And Inguinofemoral Lymphadenectomy Using Separate Groin Incisions | Gaarenstroom, Kn; Kenter, Gg; Trimbos, Jb; Agous, I; Amant, F; Peters, Aaw; Vergote, I | International Journal Of Gynecological Cancer | 2003 | 208 | 10.95 |
| **86** | Treatment Of Lymphedema Of The Arms And Legs With 5,6-Benzo-[Alpha]-Pyrone | Casleysmith, Jr; Morgan, Rg; Piller, Nb | New England Journal Of Medicine | 1993 | 208 | 7.17 |
| **87** | The Risk Of Developing Arm Lymphedema Among Breast Cancer Survivors: A Meta-Analysis Of Treatment Factors | Tsai, Rebecca J.; Dennis, Leslie K.; Lynch, Charles F.; Snetselaar, Linda G.; Zamba, Gideon K. D.; Scott-Conner, Carol | Annals Of Surgical Oncology | 2009 | 207 | 15.92 |
| **88** | Materials And Designs For Wireless Epidermal Sensors Of Hydration And Strain | Huang, Xian; Liu, Yuhao; Cheng, Huanyu; Shin, Woo-Jung; Fan, Jonathan A.; Liu, Zhuangjian; Lu, Ching-Jui; Kong, Gil-Woo; Chen, Kaile; Patnaik, Dwipayan; Lee, Sang-Heon; Hage-Ali, Sami; Huang, Yonggang; Rogers, John A. | Advanced Functional Materials | 2014 | 205 | 25.63 |
| **89** | Upper-Body Morbidity After Breast Cancer | Hayes, Sandra C.; Johansson, Karin; Stout, Nicole L.; Prosnitz, Robert; Armer, Jane M.; Gabram, Sheryl; Schmitz, Kathryn H. | Cancer | 2012 | 205 | 20.5 |
| **90** | Estimating The Population Burden Of Lymphedema | Rockson, Stanley G.; Rivera, Kahealani K. | Lymphatic Continuum Revisited | 2008 | 203 | 14.5 |
| **91** | Effect Of Upper Extremity Exercise On Secondary Lymphedema In Breast Cancer Patients: A Pilot Study | Mckenzie, Dc; Kalda, Al | Journal Of Clinical Oncology | 2003 | 201 | 10.58 |
| **92** | Quality Of Life Of Breast Cancer Patients With Lymphedema | Velanovich, V; Szymanski, V | American Journal Of Surgery | 1999 | 200 | 8.7 |
| **93** | Effective Treatment Of Lymphedema Of The Extremities | Ko, Dsc; Lerner, R; Klose, G; Cosimi, Ab | Archives Of Surgery | 1998 | 199 | 8.29 |
| **94** | Interstitial Flow As A Guide For Lymphangiogenesis | Boardman, Kc; Swartz, Ma | Circulation Research | 2003 | 199 | 10.47 |
| **95** | The Forkhead Transcription Factors, Foxc1 And Foxc2, Are Required For Arterial Specification And Lymphatic Sprouting During Vascular Development | Seo, Seungwoon; Fujita, Hideo; Nakano, Atsushi; Kang, Myengmo; Duarte, Antonio; Kume, Tsutomu | Developmental Biology | 2006 | 198 | 12.38 |
| **96** | Randomized Controlled Trial Of Weight Training And Lymphedema In Breast Cancer Survivors | Ahmed, Rehana L.; Thomas, William; Yee, Douglas; Schmitz, Kathryn H. | Journal Of Clinical Oncology | 2006 | 198 | 12.38 |
| **97** | Relapse And Morbidity In Patients Undergoing Sentinel Lymph Node Biopsy Alone Or With Axillary Dissection For Breast Cancer | Blanchard, Dk; Donohue, Jh; Reynolds, C; Grant, Cs | Archives Of Surgery | 2003 | 197 | 10.37 |
| **98** | Validation Of An Optoelectronic Limb Volumeter (Perometer(R)) | Stanton, Awb; Northfield, Jw; Holroyd, B; Mortimer, Ps; Levick, Jr | Lymphology | 1997 | 197 | 7.88 |
| **99** | Lymphedema - Evaluation Of Qualitative And Quantitative Lymphoscintigraphy In 238 Patients | Weissleder, H; Weissleder, R | Radiology | 1988 | 197 | 5.79 |
| **100** | Quality Of Life And A Symptom Cluster Associated With Breast Cancer Treatment-Related Lymphedema | Ridner, Sh | Supportive Care In Cancer | 2005 | 195 | 11.47 |

**Supplementary Table S3 | High-frequency keywords in publications on lymphedema(≥10 times)**

| Rank | | Frequency | Keywords | Rank | Frequency | | Keywords |
| --- | --- | --- | --- | --- | --- | --- | --- |
| 1 | 2184 | | Lymphedema | 132 | 22 | Post Mastectomy Complications | |
| 2 | 789 | | Breast Cancer | 133 | 22 | Randomized Controlled Trial | |
| 3 | 308 | | Health-Related Quality Of Life (HRQOL) | 134 | 22 | Tissue Fluid | |
| 4 | 179 | | Lymphscintigraphy | 135 | 21 | Body Mass Index | |
| 5 | 219 | | Lymphovenous Anastomosis | 136 | 21 | Circumference Measurement | |
| 6 | 165 | | Lymphangiogenesis | 137 | 21 | Manual Lymphatic Massage | |
| 7 | 164 | | Breast Cancer-Related Lymphedema(BCLR) | 138 | 21 | Measurement | |
| 8 | 137 | | Radiation Therapy | 139 | 23 | Psychological Distress | |
| 9 | 122 | | Breast Neoplasms | 140 | 20 | Arm | |
| 10 | 123 | | Physical Exercise | 141 | 20 | Coumarin | |
| 11 | 125 | | Lymphatics | 142 | 20 | Metastasis | |
| 12 | 113 | | Microsurgery | 143 | 24 | Nursing | |
| 13 | 101 | | Edema | 144 | 20 | Pleural Effusion | |
| 14 | 99 | | Rehabilitation | 145 | 20 | Upper and Lower Extremity | |
| 15 | 97 | | Lymphadenectomy | 146 | 19 | Compression Bandaging | |
| 16 | 95 | | Cervical Cancer | 147 | 19 | Incidence | |
| 17 | 98 | | Treatment | 148 | 19 | Massive Localized Lymphedema | |
| 18 | 100 | | Bioimpedance | 149 | 19 | Patient-Reported Outcomes | |
| 19 | 94 | | Axillary Lymph Node Dissection(ALND) | 150 | 19 | Venous Insufficiency | |
| 20 | 94 | | Lymphatic Filariasis | 151 | 19 | Women | |
| 21 | 93 | | Sentinel Lymph Node Biopsy | 152 | 20 | Wuchereria Bancrofti | |
| 22 | 92 | | Cancer | 153 | 18 | Adipose Tissue | |
| 23 | 90 | | Lymphatic Vessels | 154 | 18 | Hennekam's Syndrome | |
| 24 | 86 | | Manual Lymphatic Drainage(MLD) | 155 | 21 | Sirolimus | |
| 25 | 84 | | Primary Lymphoedema | 156 | 18 | Surgical Treatment | |
| 26 | 91 | | Vascularized Lymph Node Transfer (VLNT) | 157 | 18 | Surveys and Questionnaires | |
| 27 | 84 | | Complex Decongestive Therapy | 158 | 18 | Acupuncture、Traditional Chinese Medicine | |
| 28 | 77 | | Sentinel Lymph Node | 159 | 17 | Adverse Effects | |
| 29 | 78 | | Lower Limb Lymphedema | 160 | 17 | Animal Model | |
| 30 | 77 | | VEGF-C | 161 | 17 | Function | |
| 31 | 72 | | Obesity | 162 | 17 | Lymphatic Surgery | |
| 32 | 72 | | Endometrial Cancer | 163 | 17 | Prognosis | |
| 33 | 71 | | Magnetic Resonance Imaging | 164 | 17 | SPECT/CT | |
| 34 | 69 | | Lymph Nodes | 165 | 17 | VEGF | |
| 35 | 69 | | Secondary Lymphedema | 166 | 16 | Aging | |
| 36 | 75 | | ICG | 167 | 17 | Charles Procedure | |
| 37 | 65 | | Gynecologic Cancer | 168 | 16 | Education | |
| 38 | 63 | | Mastectomy | 169 | 16 | LEG | |
| 39 | 63 | | Surgery | 170 | 16 | Lymph Node Transplantation | |
| 40 | 63 | | Review | 171 | 17 | Lymphangioma | |
| 41 | 58 | | Vulval Cancer | 172 | 16 | Lymphatic Development | |
| 42 | 58 | | Upper Limb Lymphedema | 173 | 17 | Pelvic Lymphadenectomy | |
| 43 | 56 | | FOXC2 | 174 | 16 | Radical Hysterectomy | |
| 44 | 55 | | Lower Extremity | 175 | 16 | Rheumatoid Arthritis | |
| 45 | 54 | | Angiosarcoma | 176 | 16 | Scrotal Lymphedema | |
| 46 | 53 | | Liposuction | 177 | 16 | Toxicity | |
| 47 | 51 | | Melanoma | 178 | 18 | Traditional Chinese Medicine、Integrativ Medicine | |
| 48 | 51 | | Risk Factors | 179 | 16 | Yoga | |
| 49 | 50 | | Complications | 180 | 15 | Children | |
| 50 | 53 | | Elephantiasis | 181 | 15 | Combination Therapy | |
| 51 | 49 | | Inflammation | 182 | 15 | Deep Vein Thrombosis | |
| 52 | 49 | | Pain | 183 | 15 | Endothelial Cells | |
| 53 | 48 | | Chronic Lymphedema | 184 | 15 | FLT4 | |
| 54 | 48 | | Lymphatic Endothelial Cells | 185 | 15 | Fluorescence Imaging | |
| 55 | 48 | | Physical Activity | 186 | 15 | Immunohistochemistry | |
| 56 | 48 | | Physical Therapy | 187 | 15 | Intestinal Lymphangiectasis | |
| 57 | 47 | | Cellulitis | 188 | 15 | Kinesio Taping | |
| 58 | 50 | | Lipedema | 189 | 15 | Lymphatic Disease | |
| 59 | 47 | | Lymph Node Transfer | 190 | 15 | Ovarian Cancer | |
| 60 | 47 | | Upper Extremity | 191 | 15 | Questionnaire | |
| 61 | 46 | | Head and NeckCancer | 192 | 15 | Seroma | |
| 62 | 45 | | Neoplasms | 193 | 15 | Staging | |
| 63 | 44 | | Compression | 194 | 14 | Axillary Web Syndrome | |
| 64 | 44 | | Yellow Nail Syndrome | 195 | 14 | Disability | |
| 65 | 42 | | Diagnosis | 196 | 16 | Hemangioma | |
| 66 | 42 | | Lymphatic System | 197 | 14 | Infection | |
| 67 | 41 | | Axillary Reverse Mapping | 198 | 14 | Lymph Flow | |
| 68 | 47 | | Self-Management | 199 | 14 | Lymphedema Treatment | |
| 69 | 45 | | Lymphedema Measurement | 200 | 14 | Next Generation Sequencing (NGS) | |
| 70 | 39 | | Morbidity | 201 | 14 | Penile Cancer | |
| 71 | 37 | | Angiogenesis | 202 | 14 | Recurrence | |
| 72 | 37 | | Postoperative Complications | 203 | 14 | Swelling | |
| 73 | 36 | | Survivorship | 204 | 13 | Cancer Survivors | |
| 74 | 35 | | Fibrosis | 205 | 14 | Chorioretinal Dysplasia | |
| 75 | 35 | | ICG Lymphography | 206 | 13 | Depression | |
| 76 | 35 | | Lymphography | 207 | 13 | Elephantiasis Nostras Verrucosa | |
| 77 | 38 | | Magnetic Resonance Lymphangiography | 208 | 13 | Fatigue | |
| 78 | 39 | | GATA2 | 209 | 13 | Lymphatic Imaging | |
| 79 | 35 | | Lymphedema-Distichiasis Syndrome | 210 | 13 | Lymphatic Vasculature | |
| 80 | 34 | | Prox1 | 211 | 13 | Minimally Invasive Surgery | |
| 81 | 34 | | Radiation | 212 | 13 | Morbid Obesity | |
| 82 | 33 | | Axillary Dissection | 213 | 13 | Noonan Syndrome | |
| 83 | 33 | | Symptoms | 214 | 14 | Rat Model | |
| 84 | 36 | | Volumetry | 215 | 13 | Sarcoma | |
| 85 | 32 | | Breast Reconstruction | 216 | 13 | Side Effects | |
| 86 | 32 | | Chemotherapy | 217 | 13 | Thoracic Duct | |
| 87 | 32 | | Oncology | 218 | 13 | Turner Syndrome | |
| 88 | 32 | | Survivors | 219 | 13 | VEGF-D | |
| 89 | 31 | | Chylothorax | 220 | 13 | Vulva | |
| 90 | 31 | | Compression Therapy | 221 | 13 | Wound Healing | |
| 91 | 31 | | Milroy Disease | 222 | 12 | Breast Cancer Treatment | |
| 92 | 31 | | Tissue Dielectric Constant | 223 | 12 | Cancer Survivorship | |
| 93 | 32 | | Kaposi Sarcoma | 224 | 12 | Hereditary Lymphoedema | |
| 94 | 34 | | Low-Level Laser Therapy | 225 | 12 | Hydrocele | |
| 95 | 32 | | Mouse Model | 226 | 12 | Intervention | |
| 96 | 30 | | Reliability | 227 | 12 | Laparoscopy | |
| 97 | 30 | | VEGFR-3 | 228 | 12 | Late Effects | |
| 98 | 29 | | Breast | 229 | 12 | Lymphangitis | |
| 99 | 30 | | Intermittent Pneumatic Compression | 230 | 12 | Lymphatic Metastasis | |
| 100 | 30 | | Near-Infrared Fluorescence Imaging | 231 | 12 | Lymphocele | |
| 101 | 31 | | Plastic & Reconstructive Surgery | 232 | 12 | Management | |
| 102 | 29 | | Prevention | 233 | 12 | Paclitaxel | |
| 103 | 29 | | Ultrasound | 234 | 12 | Primary | |
| 104 | 29 | | Validity | 235 | 12 | Reconstruction | |
| 105 | 28 | | Compression Stockings | 236 | 12 | Resistance Training | |
| 106 | 28 | | Inguinal Lymphadenectomy | 237 | 12 | Scrotum | |
| 107 | 28 | | Lymphatic Drainage | 238 | 12 | Skin Water | |
| 108 | 29 | | Chronic Venous Disease | 239 | 12 | SOX18 | |
| 109 | 27 | | Lymphangiography | 240 | 12 | Transplantation | |
| 110 | 30 | | Breast Cancer Survivors | 241 | 11 | Arm Volume | |
| 111 | 26 | | Complete Decongestive Therapy | 242 | 11 | Autosomal Recessive | |
| 112 | 26 | | Lymph Node Dissection | 243 | 11 | Blue Dye | |
| 113 | 26 | | Lymphangiosarcoma | 244 | 11 | Breast Surgery | |
| 114 | 30 | | Penis | 245 | 11 | Case Report | |
| 115 | 25 | | Genetics | 246 | 11 | Development | |
| 116 | 25 | | Lymphangiectasia | 247 | 11 | Epidemiology | |
| 117 | 25 | | Lymphedema Surgery | 248 | 14 | Limprint | |
| 118 | 25 | | Stewart-Treves Syndrome | 249 | 11 | Lymphatic Function | |
| 119 | 24 | | Breast-Conserving Therapy | 250 | 11 | Lymphedema Therapy | |
| 120 | 24 | | Meta-Analysis | 251 | 11 | LYVE-1 | |
| 121 | 24 | | Prevalence | 252 | 11 | Mental Retardation | |
| 122 | 24 | | Skin | 253 | 11 | Mutation | |
| 123 | 24 | | Ultrasonography | 254 | 11 | Neoadjuvant Chemotherapy | |
| 124 | 27 | | Vascular Malformation | 255 | 11 | Physical Therapy Modalities | |
| 125 | 24 | | VEGFR3 | 256 | 11 | Prostate Cancer | |
| 126 | 23 | | Bandaging | 257 | 11 | Qualitative Research | |
| 127 | 23 | | Erysipelas | 258 | 11 | Tuberous Sclerosis Complex | |
| 128 | 23 | | Lymphatic Malformation | 259 | 11 | Uterine Cancer | |
| 129 | 24 | | Adipose-Derived Stem Cells | 260 | 11 | Water Displacement Method | |
| 130 | 22 | | Imaging | 261 | 11 | Zebrafish | |
| 131 | 22 | | Microcephaly |  |  |  | |


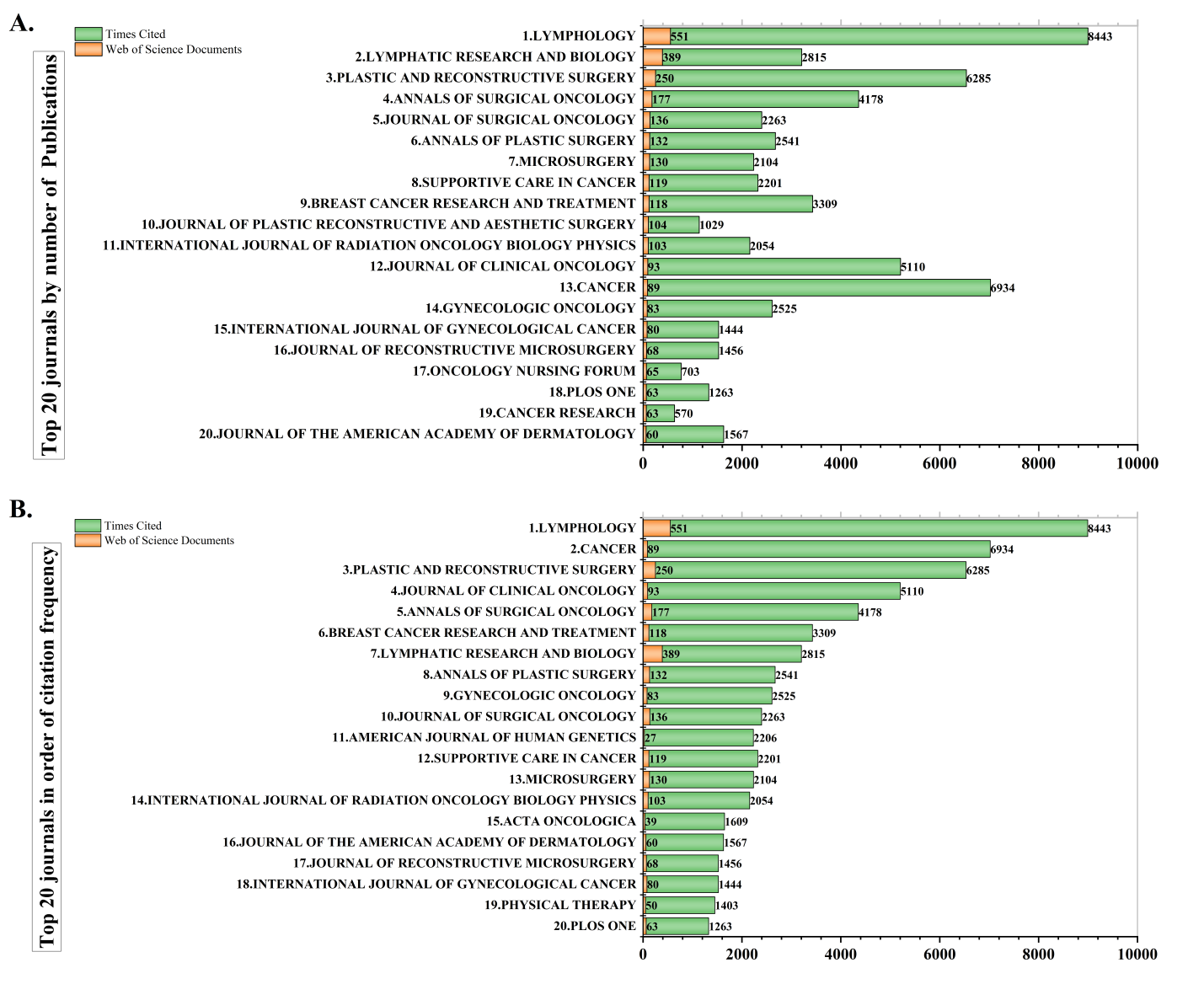


**Supplementary Figure S1 |** The top 20 posting journals and top 20 cited frequency journals. **(A)**The top 20 journals and their citation frequencies by the number of publications. **(B)**The top 20 journals and their citation frequencies by their citation frequencies.
